# Supplementary material for: Determining virus-host interactions and glycerol metabolism profiles in geographically diverse solar salterns with metagenomics
Source: PeerJ. 2017 Jan 10;5:e2844. doi: 10.7717/peerj.2844 (PMC5228507; doi:10.7717/peerj.2844)
Supplement: Figure S1 — The sections covered include profiling taxonomic and functional compositions of selected metagenomes, de novo and reference-guided CRISPR detection, mapping virus-host interactions with CRISPR spacers, comparative analysis of virus and host sequence characteristics, and assembling metagenomes and detecting halobacterial cas genes in metagenomic contigs. [file peerj-05-2844-s001.pdf]

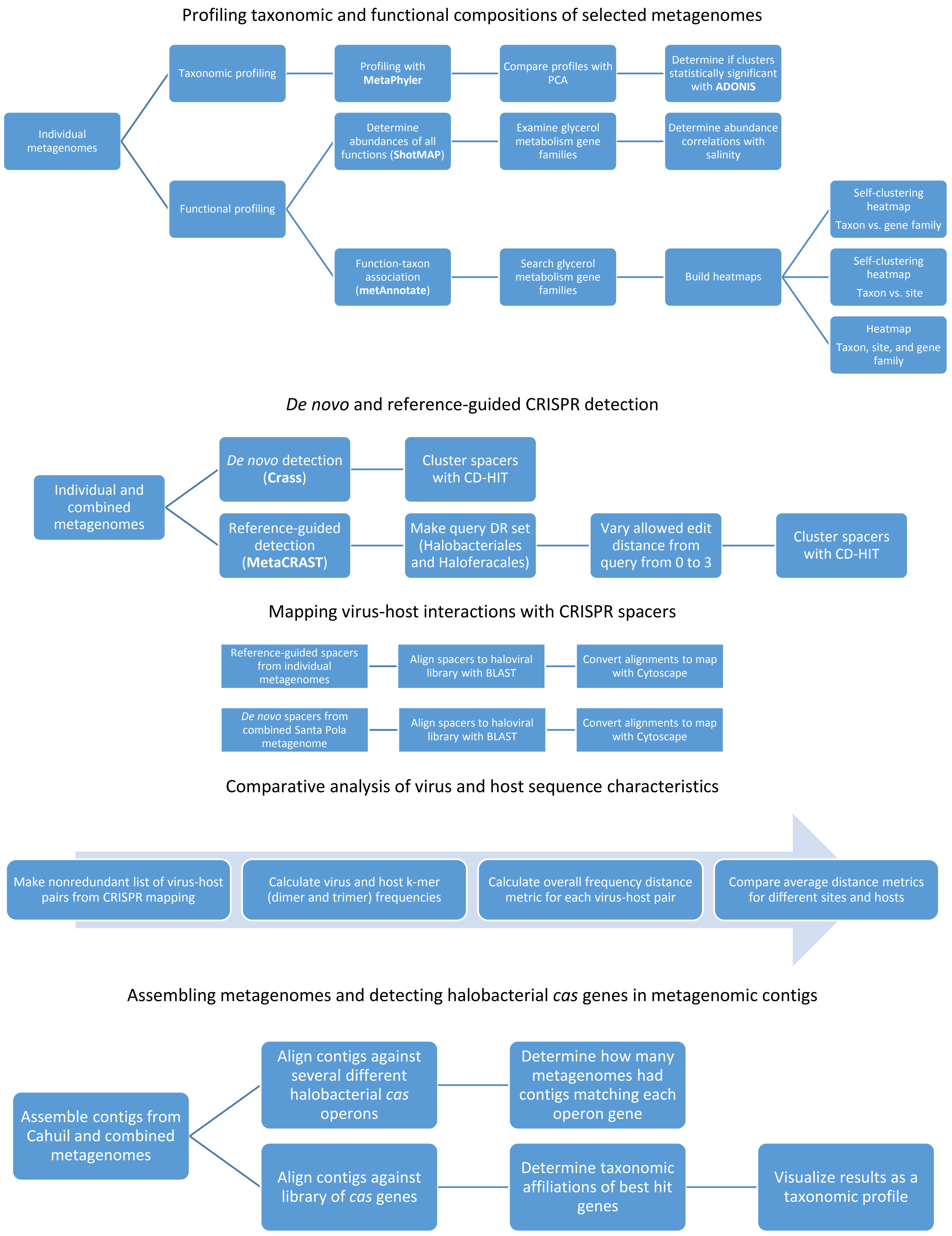
Profiling taxonomic and functional compositions of selected metagenomes
Individual metagenomes
Taxonomic profiling
Profiling with **MetaPhyler**
Compare profiles with PCA
Determine if clusters statistically significant with **ADONIS**
Functional profiling
Determine abundances of all functions (**ShotMAP**)
Examine glycerol metabolism gene families
Determine abundance correlations with salinity
Function-taxon association (**metAnnotate**)
Search glycerol metabolism gene families
Build heatmaps
Self-clustering heatmap Taxon vs. gene family
Self-clustering heatmap Taxon vs. site
Heatmap Taxon, site, and gene family
De novo and reference-guided CRISPR detection
Individual and combined metagenomes
De novo detection (**Crass**)
Cluster spacers with CD-HIT
Reference-guided detection (**MetaCRAST**)
Make query DR set (Halobacteriales and Haloferacales)
Vary allowed edit distance from query from 0 to 3
Cluster spacers with CD-HIT
Mapping virus-host interactions with CRISPR spacers
Reference-guided spacers from individual metagenomes
Align spacers to haloviral library with BLAST
Convert alignments to map with Cytoscape
De novo spacers from combined Santa Pola metagenome
Align spacers to haloviral library with BLAST
Convert alignments to map with Cytoscape
Comparative analysis of virus and host sequence characteristics
Make nonredundant list of virus-host pairs from CRISPR mapping
Calculate virus and host k-mer (dimer and trimer) frequencies
Calculate overall frequency distance metric for each virus-host pair
Compare average distance metrics for different sites and hosts
Assembling metagenomes and detecting halobacterial *cas* genes in metagenomic contigs
Assemble contigs from Cahuil and combined metagenomes
Align contigs against several different halobacterial *cas* operons
Determine how many metagenomes had contigs matching each operon gene
Align contigs against library of *cas* genes
Determine taxonomic affiliations of best hit genes
Visualize results as a taxonomic profile
