# Supplementary figures and images for: Determining virus-host interactions and glycerol metabolism profiles in geographically diverse solar salterns with metagenomics
Source: PeerJ. 2017 Jan 10;5:e2844. doi: 10.7717/peerj.2844 (PMC5228507; doi:10.7717/peerj.2844)

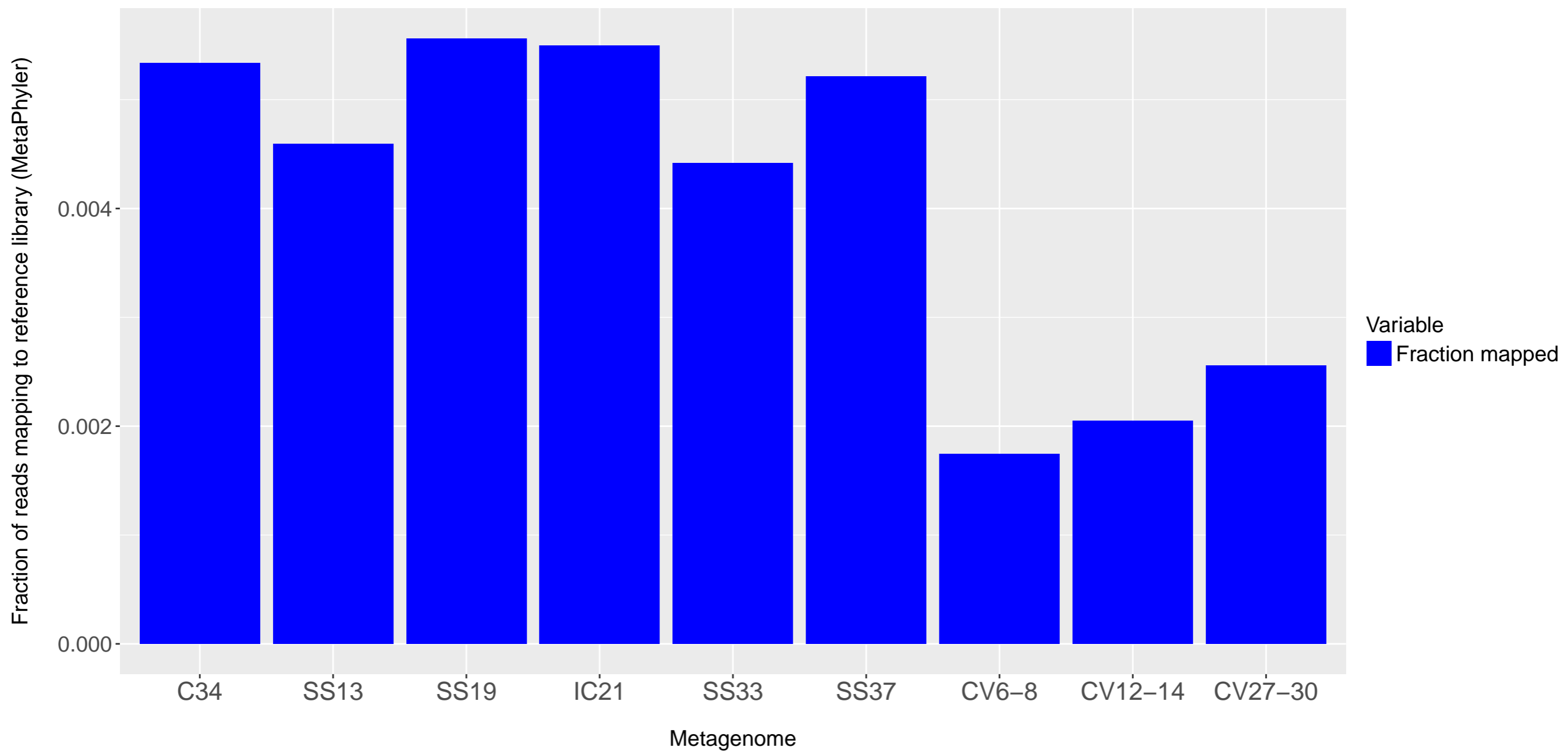

Supplement: Figure S2 [file peerj-05-2844-s002.pdf]

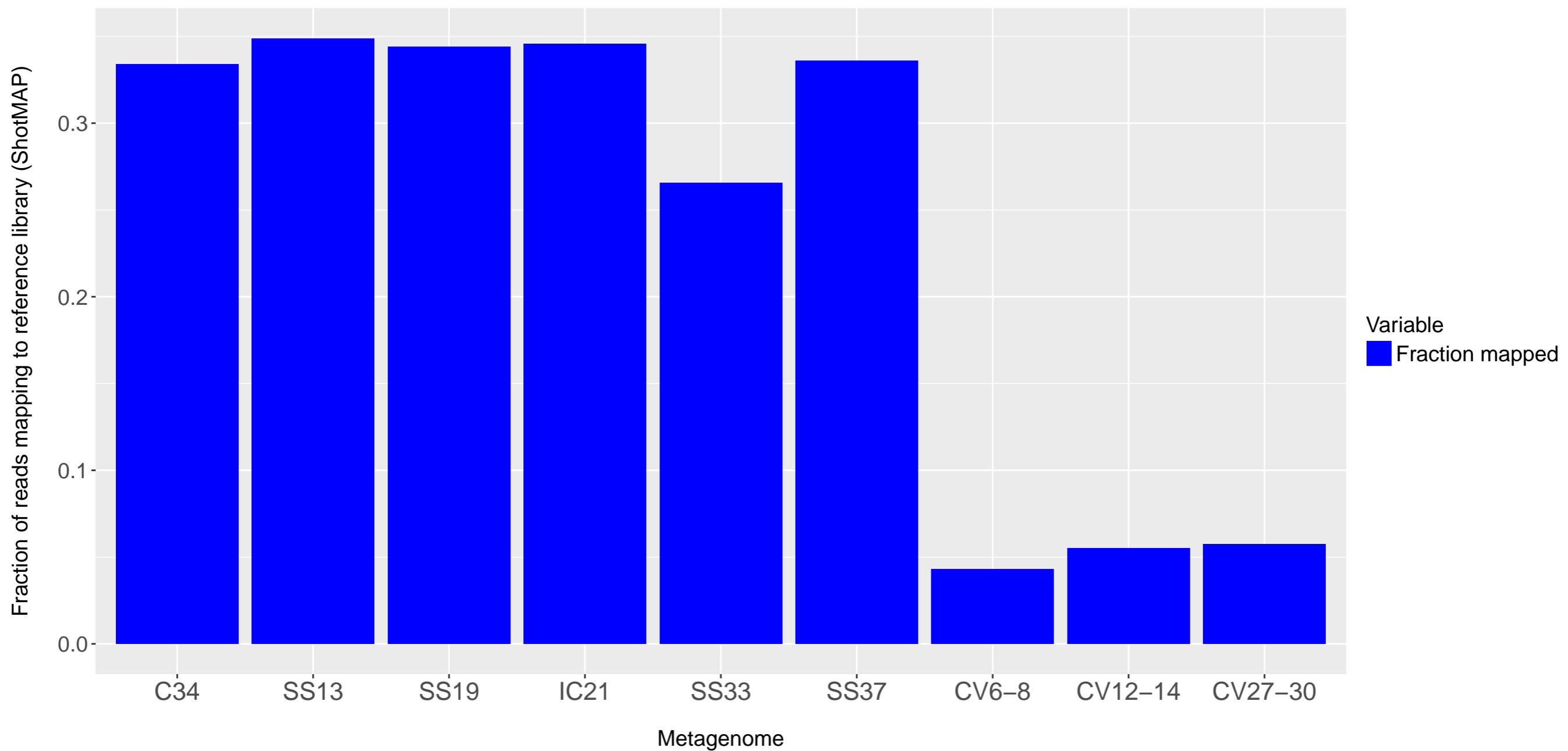

Supplement: Figure S3 [file peerj-05-2844-s003.pdf]

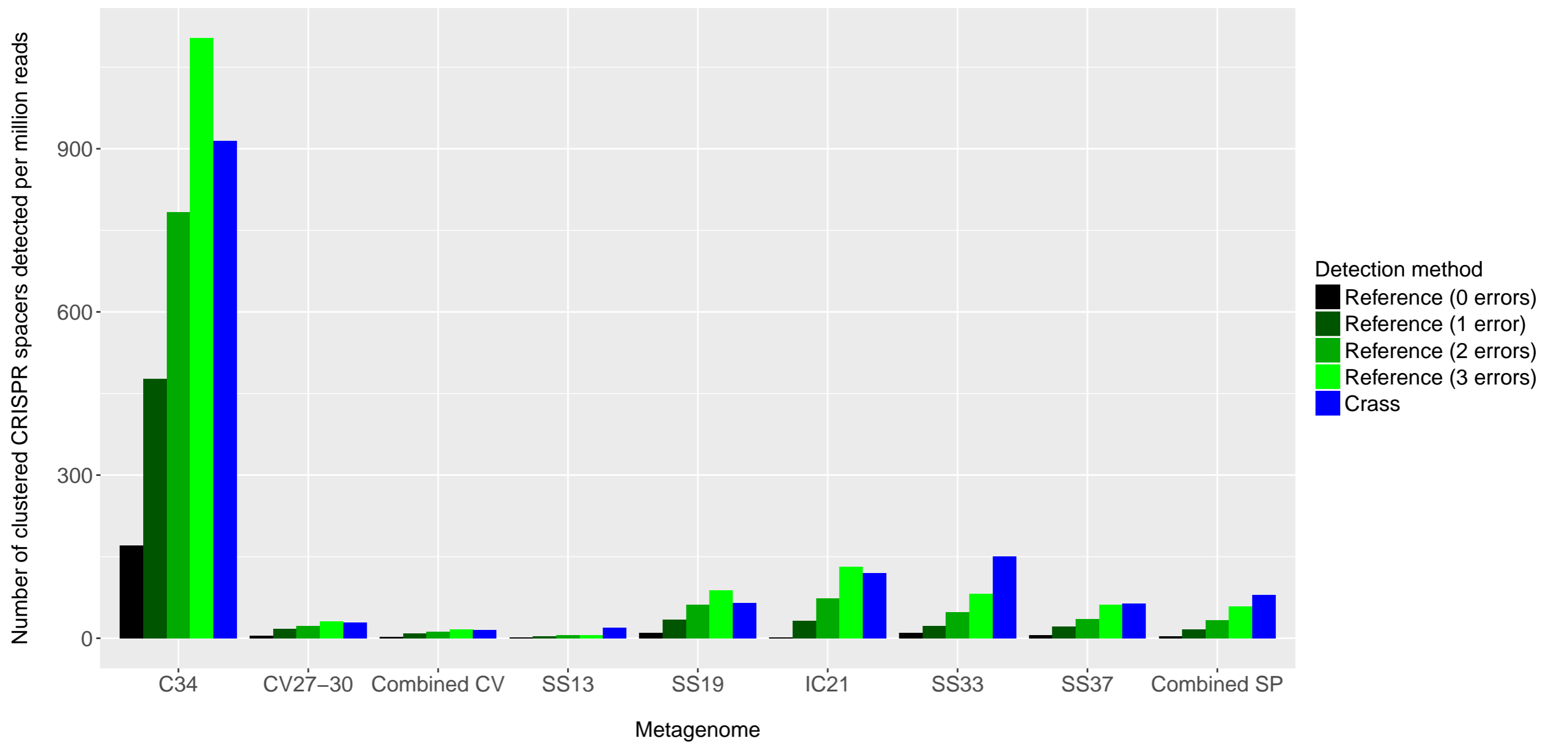

Supplement: Figure S4 — Spacers were detected either with the de novo detection method (Crass) or the reference-guided method (MetaCRAST) with corresponding maximum edit distances (from 0 to 3) described in Materials and Methods that used a query of 29 halobacterial CRISPR direct repeat sequences. All spacer counts are reported after clustering initially detected spacers with CD-HIT (with a clustering similarity threshold of 0.9). [file peerj-05-2844-s004.pdf]

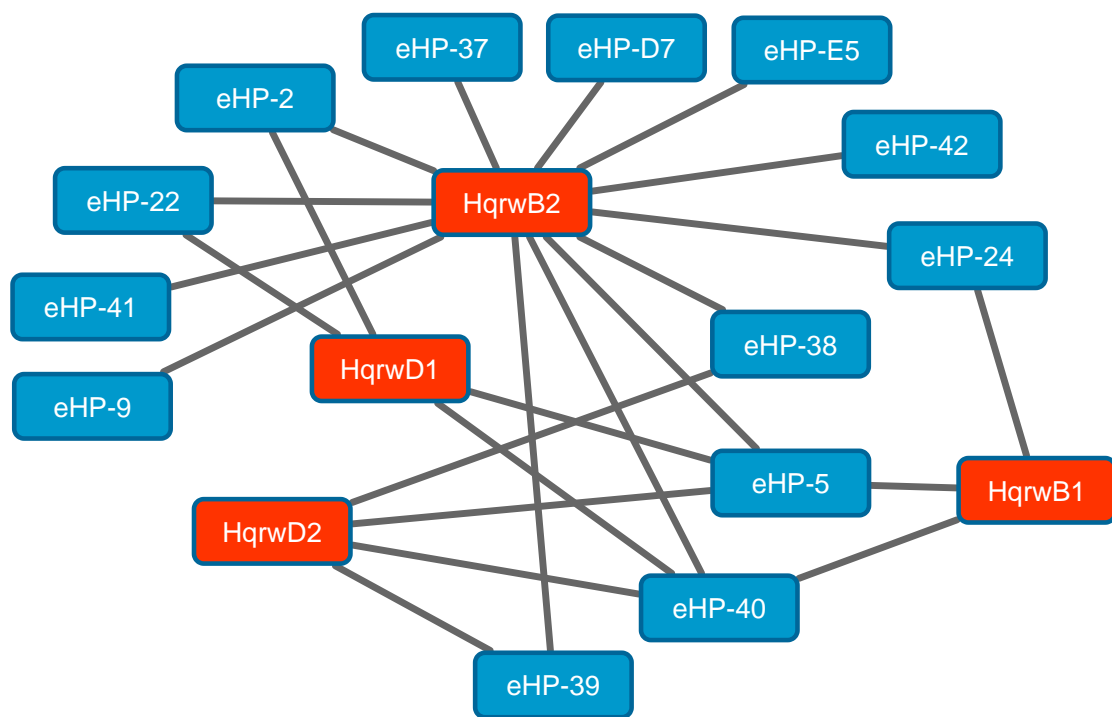

Supplement: Figure S5 — The library of haloviral genomes screened is listed in Table S1. Nodes represent either viruses or spacers, while edges represent BLAST alignments linking spacers to viruses. Viruses are marked in blue and spacers in orange. Visualization was performed with Cytoscape. [file peerj-05-2844-s005.pdf]

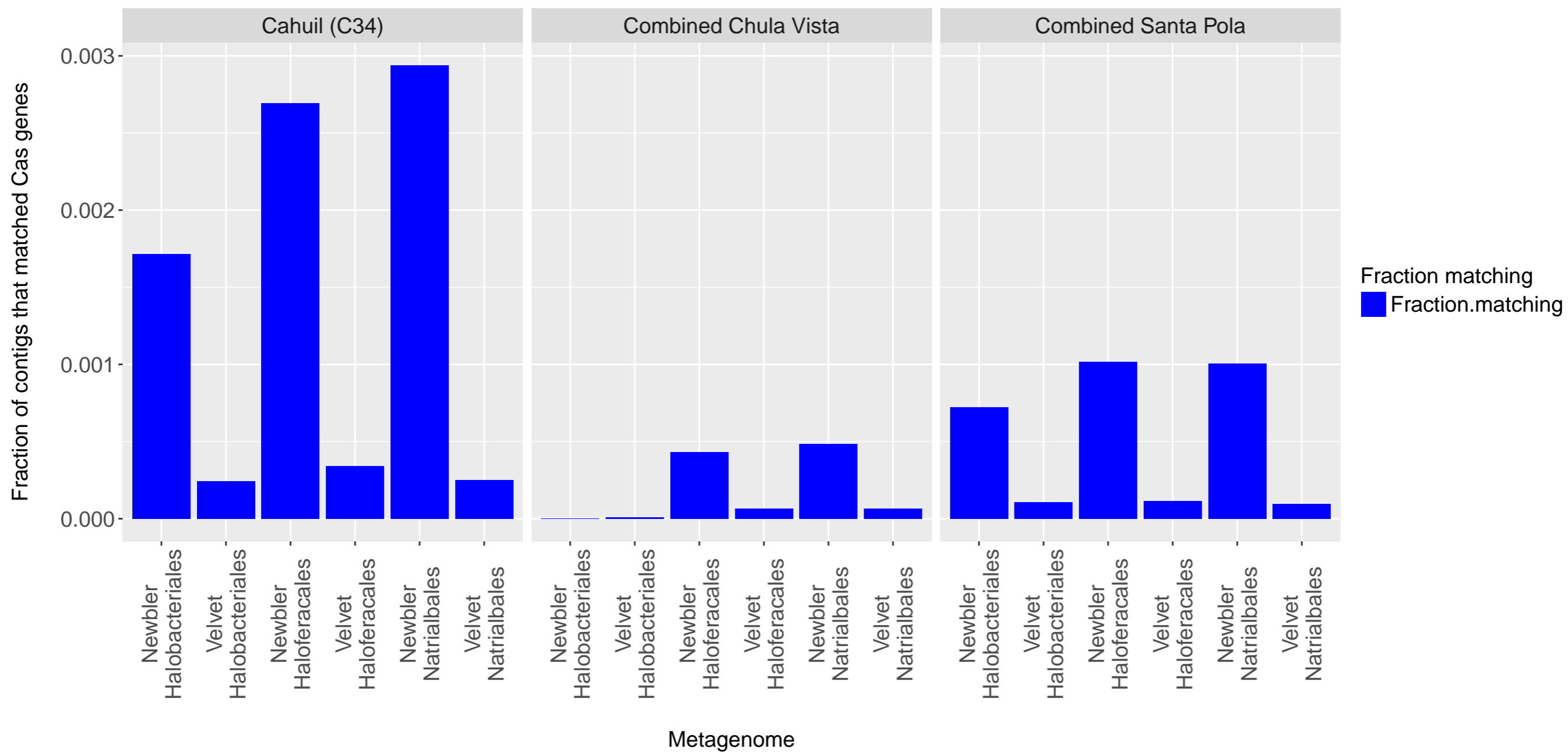

Supplement: Figure S6 [file peerj-05-2844-s006.pdf]

Proportion of all matching contigs

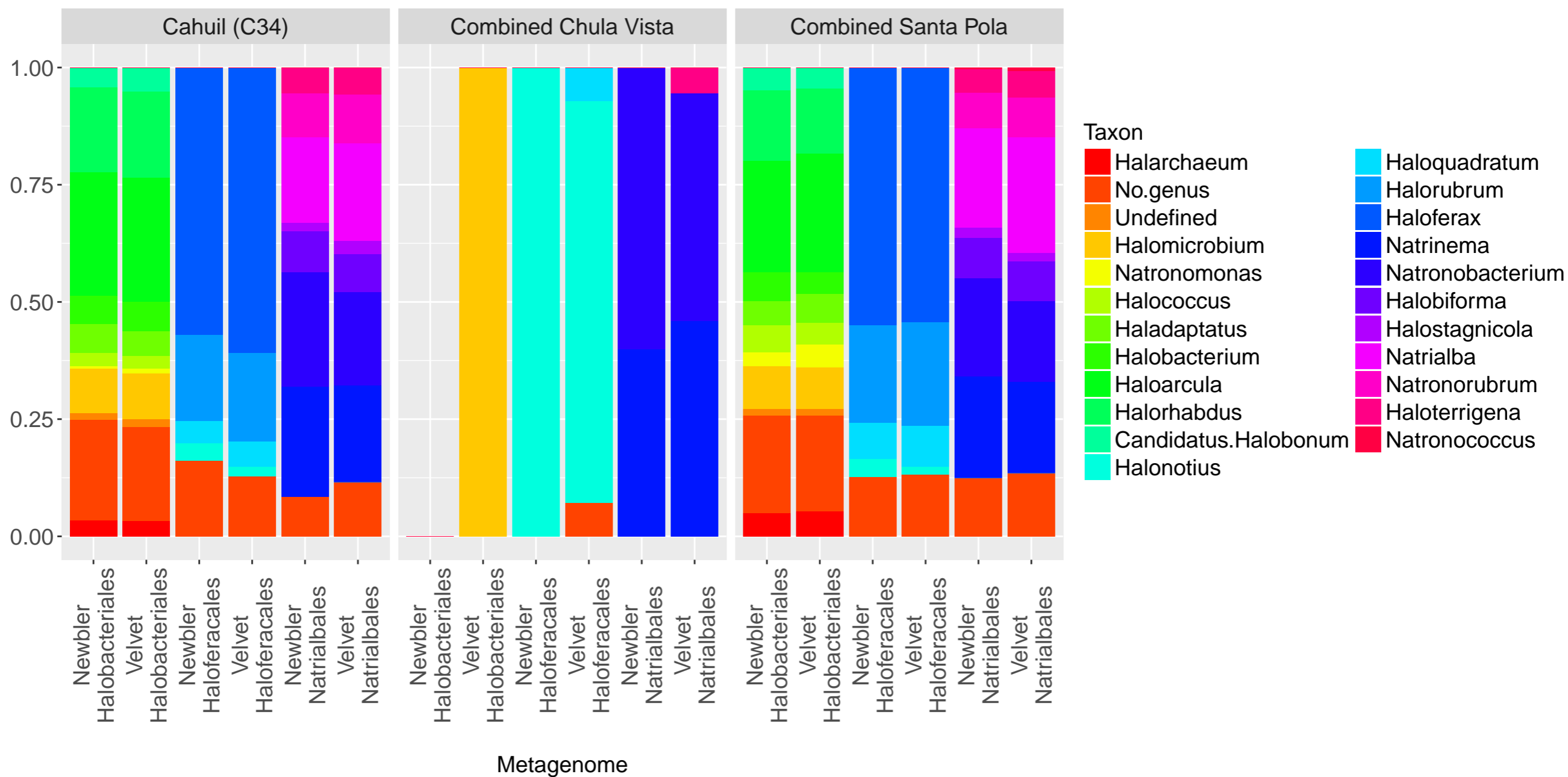

Supplement: Figure S7 — Detected contigs assembled with Newbler and Velvet were aligned against a library of cas genes from three halobacterial orders (Halobacteriales, Haloferacales, and Natrialbales). The taxonomic affiliations of all hits for each contig were tabulated into profiles for each order and assembly method. [file peerj-05-2844-s007.pdf]
