# Supplementary material for: Determining virus-host interactions and glycerol metabolism profiles in geographically diverse solar salterns with metagenomics
Source: PeerJ. 2017 Jan 10;5:e2844. doi: 10.7717/peerj.2844 (PMC5228507; doi:10.7717/peerj.2844)
Supplement: Table S1 — The library included 326 contigs more than 500 bp in length assembled from Chula Vista metaviromes (http://data.imicrobe.us/project/view/58) and the following haloviral genomes obtained from NCBI GenBank. [file peerj-05-2844-s008.docx]

Table S1: The library of haloviral genomes examined included 326 contigs more than 500 bp in length assembled from Chula Vista metaviromes (http://data.imicrobe.us/project/view/58) and the following haloviral genomes obtained from NCBI GenBank.

| GI | GB | Description |
| --- | --- | --- |
| 82492114 | DQ238866.1 | Environmental halophage 1 AAJ-2005, partial genome |
| 383398351 | JQ807263.1 | Environmental Halophage eHP-E5, partial genome |
| 383398308 | JQ807262.1 | Environmental Halophage eHP-D7, partial genome |
| 383398280 | JQ807261.1 | Environmental Halophage eHP-42, partial genome |
| 383398256 | JQ807260.1 | Environmental Halophage eHP-41, partial genome |
| 383398200 | JQ807259.1 | Environmental Halophage eHP-40, partial genome |
| 383398172 | JQ807258.1 | Environmental Halophage eHP-39, partial genome |
| 383398140 | JQ807257.1 | Environmental Halophage eHP-38, partial genome |
| 383398101 | JQ807256.1 | Environmental Halophage eHP-37, partial genome |
| 383398056 | JQ807255.1 | Environmental Halophage eHP-36, partial genome |
| 383398012 | JQ807254.1 | Environmental Halophage eHP-35, partial genome |
| 383397960 | JQ807253.1 | Environmental Halophage eHP-34, partial genome |
| 383397929 | JQ807252.1 | Environmental Halophage eHP-33, partial genome |
| 383397879 | JQ807251.1 | Environmental Halophage eHP-32, partial genome |
| 383397836 | JQ807250.1 | Environmental Halophage eHP-31, partial genome |
| 383397780 | JQ807249.1 | Environmental Halophage eHP-30, partial genome |
| 383397746 | JQ807248.1 | Environmental Halophage eHP-29, partial genome |
| 383397713 | JQ807247.1 | Environmental Halophage eHP-28, partial genome |
| 383397672 | JQ807246.1 | Environmental Halophage eHP-27, partial genome |
| 383397640 | JQ807245.1 | Environmental Halophage eHP-25, partial genome |
| 383397593 | JQ807244.1 | Environmental Halophage eHP-24, partial genome |
| 383397546 | JQ807243.1 | Environmental Halophage eHP-23, partial genome |
| 383397498 | JQ807242.1 | Environmental Halophage eHP-22, partial genome |
| 383397450 | JQ807241.1 | Environmental Halophage eHP-20, partial genome |
| 383397419 | JQ807240.1 | Environmental Halophage eHP-19, partial genome |
| 383397370 | JQ807239.1 | Environmental Halophage eHP-18, partial genome |
| 383397340 | JQ807238.1 | Environmental Halophage eHP-17, partial genome |
| 383397303 | JQ807237.1 | Environmental Halophage eHP-16, partial genome |
| 383397240 | JQ807236.1 | Environmental Halophage eHP-15, partial genome |
| 383397189 | JQ807235.1 | Environmental Halophage eHP-14, partial genome |
| 383397132 | JQ807234.1 | Environmental Halophage eHP-13, partial genome |
| 383397082 | JQ807233.1 | Environmental Halophage eHP-12, partial genome |
| 383397053 | JQ807232.1 | Environmental Halophage eHP-11, partial genome |
| 383397015 | JQ807231.1 | Environmental Halophage eHP-10, partial genome |
| 383396977 | JQ807230.1 | Environmental Halophage eHP-9, partial genome |
| 383396921 | JQ807229.1 | Environmental Halophage eHP-8, partial genome |
| 383396883 | JQ807228.1 | Environmental Halophage eHP-7, partial genome |
| 383396834 | JQ807227.1 | Environmental Halophage eHP-6, partial genome |
| 383396790 | JQ807226.1 | Environmental Halophage eHP-5, partial genome |
| 383396741 | JQ807225.1 | Environmental Halophage eHP-4, partial genome |
| 383396711 | JQ807224.1 | Environmental Halophage eHP-3, partial genome |
| 383396670 | JQ807223.1 | Environmental Halophage eHP-2, partial genome |
| 383396626 | JQ807222.1 | Environmental Halophage eHP-1, partial genome |
| 18138397 | NC_003345.1 | Halorubrum phage HF2, complete genome |
| 18000338 | AF222060.1 | Halorubrum phage HF2, complete genome |
| 505833397 | KC292029.1 | Halovirus HCTV-1, complete genome |
| 505833276 | KC292028.1 | Halovirus HCTV-2, complete genome |
| 505833087 | KC292027.1 | Halovirus HCTV-5, complete genome |
| 32346393 | AY190604.1 | Halovirus HF1, complete genome |
| 505832621 | KC292026.1 | Halovirus HGTV-1, complete genome |
| 505832536 | KC292025.1 | Halovirus HHTV-1, complete genome |
| 505832447 | KC292024.1 | Halovirus HHTV-2, complete genome |
| 505832373 | KC292023.1 | Halovirus HRTV-4, complete genome |
| 505832254 | KC292022.1 | Halovirus HRTV-5, complete genome |
| 505832148 | KC292021.1 | Halovirus HRTV-7, complete genome |
| 505832020 | KC292020.1 | Halovirus HRTV-8, complete genome |
| 441462254 | KC117378.1 | Halovirus HSTV-1, complete genome |
| 441461976 | KC117376.1 | Halovirus HSTV-2, complete genome |
| 441462080 | KC117377.1 | Halovirus HVTV-1, complete genome |
| 442736110 | KC252997.1 | Halovirus PH1, complete genome |
| 660731908 | KF771641.1 | Halovirus VNH-1 genomic sequence |
| 33338237 | AF191796.1 | His1 virus, complete genome |
| 78172403 | AF191797.1 | His2 virus, complete genome |
| 751367590 | AKVG01000001.1 | Hypersaline lake metagenome LTV1, whole genome shotgun sequence |
| 751367589 | AKVG01000002.1 | Hypersaline lake metagenome LTV2, whole genome shotgun sequence |
| 751367588 | AKVG01000003.1 | Hypersaline lake metagenome LTVLE3, whole genome shotgun sequence |
| 751367587 | AKVG01000004.1 | Hypersaline lake metagenome LTV4, whole genome shotgun sequence |
| 751367586 | AKVG01000005.1 | Hypersaline lake metagenome LTV5, whole genome shotgun sequence |
| 751367585 | AKVG01000006.1 | Hypersaline lake metagenome LTV6, whole genome shotgun sequence |
| 751367584 | AKVG01000007.1 | Hypersaline lake metagenome LTV7, whole genome shotgun sequence |
| 751367583 | AKVG01000008.1 | Hypersaline lake metagenome LTVcontig54590, whole genome shotgun sequence |
| 751367582 | AKVG01000009.1 | Hypersaline lake metagenome LTVcontig02397, whole genome shotgun sequence |
| 751367581 | AKVG01000010.1 | Hypersaline lake metagenome LTVcontig822605, whole genome shotgun sequence |
| 751367580 | AKVG01000011.1 | Hypersaline lake metagenome LTVcontig823649, whole genome shotgun sequence |
| 751367579 | AKVG01000012.1 | Hypersaline lake metagenome LTVcontig823798, whole genome shotgun sequence |
| 751367578 | AKVG01000013.1 | Hypersaline lake metagenome LTVcontig824981, whole genome shotgun sequence |
| 751367577 | AKVG01000014.1 | Hypersaline lake metagenome LTVcontig3968333, whole genome shotgun sequence |
| 751367576 | AKVG01000015.1 | Hypersaline lake metagenome LTVcontig3969208, whole genome shotgun sequence |
| 751367575 | AKVG01000016.1 | Hypersaline lake metagenome LTVcontig999000, whole genome shotgun sequence |
| 751367574 | AKVG01000017.1 | Hypersaline lake metagenome LTVcontig999004, whole genome shotgun sequence |
| 751367573 | AKVG01000018.1 | Hypersaline lake metagenome LTVcontig998975, whole genome shotgun sequence |
| 751367572 | AKVG01000019.1 | Hypersaline lake metagenome LTVcontig999006, whole genome shotgun sequence |
| 751367571 | AKVG01000020.1 | Hypersaline lake metagenome LTVcontig1100059, whole genome shotgun sequence |
| 751367570 | AKVG01000021.1 | Hypersaline lake metagenome LTVcontig1100028, whole genome shotgun sequence |
| 751367569 | AKVG01000022.1 | Hypersaline lake metagenome LTVcontig1013670, whole genome shotgun sequence |
| 751367568 | AKVG01000023.1 | Hypersaline lake metagenome LTVcontig1013956, whole genome shotgun sequence |
| 751367567 | AKVG01000024.1 | Hypersaline lake metagenome LTVcontig1013803, whole genome shotgun sequence |
| 751367566 | AKVG01000025.1 | Hypersaline lake metagenome LTVcontig1013342, whole genome shotgun sequence |
| 751367565 | AKVG01000026.1 | Hypersaline lake metagenome LTVcontig1013519, whole genome shotgun sequence |
| 751367564 | AKVG01000027.1 | Hypersaline lake metagenome LTVcontig224955, whole genome shotgun sequence |
| 751367563 | AKVG01000028.1 | Hypersaline lake metagenome LTVcontig1012758, whole genome shotgun sequence |
| 751367562 | AKVG01000029.1 | Hypersaline lake metagenome LTVcontig1012403, whole genome shotgun sequence |
| 751367561 | AKVG01000030.1 | Hypersaline lake metagenome LTVcontig761242, whole genome shotgun sequence |
| 751367560 | AKVG01000031.1 | Hypersaline lake metagenome LTVcontig1014414, whole genome shotgun sequence |
| 751367559 | AKVG01000032.1 | Hypersaline lake metagenome LTVcontig1015495, whole genome shotgun sequence |
| 751367558 | AKVG01000033.1 | Hypersaline lake metagenome LTVcontig1015890, whole genome shotgun sequence |
| 751367557 | AKVG01000034.1 | Hypersaline lake metagenome LTVcontig1013394, whole genome shotgun sequence |
| 751367556 | AKVG01000035.1 | Hypersaline lake metagenome LTVcontig1015952, whole genome shotgun sequence |
