# Supplementary material for: Determining virus-host interactions and glycerol metabolism profiles in geographically diverse solar salterns with metagenomics
Source: PeerJ. 2017 Jan 10;5:e2844. doi: 10.7717/peerj.2844 (PMC5228507; doi:10.7717/peerj.2844)
Supplement: Table S2 [file peerj-05-2844-s009.docx]

Table S2: Sequence assembly statistics for three different metagenomes and two different assembly algorithms.

|  | Cahuil | | Combined Santa Pola | | Combined Chula Vista | |
| --- | --- | --- | --- | --- | --- | --- |
| Assembler | Newbler | Velvet | Newbler | Velvet | Newbler | Velvet |
| N50 value | 881* | 205 | 996* | 106 | 882* | 102 |
| Average contig length (100 bp cutoff) | 687 | 239 | 709 | 200 | 315 | 152 |
| Number of contigs (100 bp cutoff) | 8,167 | 140,071 | 156,214 | 1,639,903 | 18,519 | 75,046 |
