# Supplementary material for: Determining virus-host interactions and glycerol metabolism profiles in geographically diverse solar salterns with metagenomics
Source: PeerJ. 2017 Jan 10;5:e2844. doi: 10.7717/peerj.2844 (PMC5228507; doi:10.7717/peerj.2844)
Supplement: Table S3 [file peerj-05-2844-s010.docx]

Table S3: Alignments of combined Santa Pola CRISPR direct repeats identified with Crass against a library of taxonomically annotated direct repeat sequences obtained from CRISPRdb.

| Crass DR | Taxonomic affiliation of aligned DR | Percent identity | Alignment length | E-value | Score |
| --- | --- | --- | --- | --- | --- |
| G2DR1 | Natronomonas pharaonis DSM 2160 | 100 | 28 | 2.00E-11 | 56 |
| G2DR1 | Natronomonas pharaonis DSM 2160 | 100 | 28 | 2.00E-11 | 56 |
| G2DR1 | Haloferax volcanii DS2 | 100 | 28 | 2.00E-11 | 56 |
| G2DR1 | Halogeometricum borinquense DSM 11551 | 100 | 26 | 2.00E-10 | 52 |
| G2DR1 | Natrinema sp. J7-2 | 96.43 | 28 | 4.00E-09 | 48.1 |
| G2DR1 | Halophilic archaeon DL31 | 96.43 | 28 | 4.00E-09 | 48.1 |
| G2DR1 | Haloarcula hispanica ATCC 33960 | 96.43 | 28 | 4.00E-09 | 48.1 |
| G2DR1 | Haloarcula hispanica N601 | 96.43 | 28 | 4.00E-09 | 48.1 |
| G2DR1 | Natronobacterium gregoryi SP2 | 96.43 | 28 | 4.00E-09 | 48.1 |
| G2DR1 | Haloferax volcanii DS2 | 96.43 | 28 | 4.00E-09 | 48.1 |
| G2DR1 | Haloferax volcanii DS2 | 96.43 | 28 | 4.00E-09 | 48.1 |
| G2DR1 | Haloarcula marismortui ATCC 43049 | 96.43 | 28 | 4.00E-09 | 48.1 |
| G2DR1 | Halomicrobium mukohataei DSM 12286 | 92.86 | 28 | 9.00E-07 | 40.1 |
| G2DR1 | Halomicrobium mukohataei DSM 12286 | 92.86 | 28 | 9.00E-07 | 40.1 |
| G2DR1 | Halorhabdus tiamatea SARL4B | 92.86 | 28 | 9.00E-07 | 40.1 |
| G2DR1 | Halorhabdus utahensis DSM 12940 | 92.86 | 28 | 9.00E-07 | 40.1 |
| G7DR1 | Dinoroseobacter shibae DFL 12 | 96.55 | 29 | 1.00E-09 | 50.1 |
| G7DR1 | Methylococcus capsulatus str. Bath | 96.43 | 28 | 4.00E-09 | 48.1 |
| G7DR1 | Rhodothermus marinus DSM 4252 | 100 | 20 | 9.00E-07 | 40.1 |
| G7DR1 | Alkalilimnicola ehrlichii MLHE-1 | 92.86 | 28 | 9.00E-07 | 40.1 |
| G37DR1 | Haloarcula hispanica ATCC 33960 | 93.33 | 30 | 6.00E-08 | 44.1 |
| G37DR1 | Haloarcula hispanica N601 | 93.33 | 30 | 6.00E-08 | 44.1 |
| G37DR1 | Natronobacterium gregoryi SP2 | 93.33 | 30 | 6.00E-08 | 44.1 |
| G38DR1 | Halorubrum lacusprofundi ATCC 49239 | 100 | 25 | 1.00E-09 | 50.1 |
| G38DR1 | Halophilic archaeon DL31 | 96 | 25 | 4.00E-07 | 42.1 |
| G40DR1 | Haloarcula hispanica ATCC 33960 | 93.33 | 30 | 7.00E-08 | 44.1 |
| G40DR1 | Haloarcula hispanica N601 | 93.33 | 30 | 7.00E-08 | 44.1 |
| G40DR1 | Natronobacterium gregoryi SP2 | 93.33 | 30 | 7.00E-08 | 44.1 |
| G42DR1 | Natronomonas pharaonis DSM 2160 | 100 | 30 | 1.00E-12 | 60 |
| G42DR1 | Natronomonas pharaonis DSM 2160 | 100 | 30 | 1.00E-12 | 60 |
| G42DR1 | Haloferax volcanii DS2 | 100 | 30 | 1.00E-12 | 60 |
| G42DR1 | Natrinema sp. J7-2 | 96.67 | 30 | 3.00E-10 | 52 |
| G42DR1 | Halophilic archaeon DL31 | 96.67 | 30 | 3.00E-10 | 52 |
| G42DR1 | Haloarcula hispanica ATCC 33960 | 96.67 | 30 | 3.00E-10 | 52 |
| G42DR1 | Haloarcula hispanica N601 | 96.67 | 30 | 3.00E-10 | 52 |
| G42DR1 | Natronobacterium gregoryi SP2 | 96.67 | 30 | 3.00E-10 | 52 |
| G42DR1 | Halogeometricum borinquense DSM 11551 | 100 | 26 | 3.00E-10 | 52 |
| G42DR1 | Haloferax volcanii DS2 | 96.67 | 30 | 3.00E-10 | 52 |
| G42DR1 | Haloferax volcanii DS2 | 96.67 | 30 | 3.00E-10 | 52 |
| G42DR1 | Haloarcula marismortui ATCC 43049 | 96.67 | 30 | 3.00E-10 | 52 |
| G42DR1 | Halomicrobium mukohataei DSM 12286 | 93.33 | 30 | 6.00E-08 | 44.1 |
| G42DR1 | Halomicrobium mukohataei DSM 12286 | 93.33 | 30 | 6.00E-08 | 44.1 |
| G42DR1 | Halorhabdus tiamatea SARL4B | 93.33 | 30 | 6.00E-08 | 44.1 |
| G42DR1 | Halorhabdus utahensis DSM 12940 | 93.33 | 30 | 6.00E-08 | 44.1 |
| G42DR1 | Natrialba magadii ATCC 43099 | 93.1 | 29 | 3.00E-07 | 42.1 |
| G43DR1 | Natronomonas pharaonis DSM 2160 | 93.33 | 30 | 6.00E-08 | 44.1 |
| G43DR1 | Natronomonas pharaonis DSM 2160 | 93.33 | 30 | 6.00E-08 | 44.1 |
| G43DR1 | Haloferax volcanii DS2 | 93.33 | 30 | 6.00E-08 | 44.1 |
| G57DR1 | Saccharomonospora viridis DSM 43017 | 100 | 23 | 1.00E-08 | 46.1 |
| G57DR1 | Methylococcus capsulatus str. Bath | 100 | 23 | 1.00E-08 | 46.1 |
| G59DR1 | Geobacter uraniireducens Rf4 | 100 | 25 | 1.00E-09 | 50.1 |
| G59DR1 | Komagataeibacter medellinensis NBRC 3288 | 96 | 25 | 3.00E-07 | 42.1 |
| G59DR1 | Gluconacetobacter diazotrophicus PA1 5 | 96 | 25 | 3.00E-07 | 42.1 |
| G59DR1 | Gluconacetobacter diazotrophicus PA1 5 | 96 | 25 | 3.00E-07 | 42.1 |
| G82DR1 | Natrinema sp. J7-2 | 96.67 | 30 | 3.00E-10 | 52 |
| G82DR1 | Natrinema sp. J7-2 | 100 | 26 | 3.00E-10 | 52 |
| G82DR1 | Haloarcula marismortui ATCC 43049 | 96.67 | 30 | 3.00E-10 | 52 |
| G82DR1 | Halophilic archaeon DL31 | 93.33 | 30 | 7.00E-08 | 44.1 |
| G82DR1 | Halomicrobium mukohataei DSM 12286 | 100 | 22 | 7.00E-08 | 44.1 |
| G82DR1 | Halomicrobium mukohataei DSM 12286 | 100 | 22 | 7.00E-08 | 44.1 |
| G82DR1 | Halorhabdus tiamatea SARL4B | 93.33 | 30 | 7.00E-08 | 44.1 |
| G82DR1 | Halorhabdus utahensis DSM 12940 | 93.33 | 30 | 7.00E-08 | 44.1 |
| G82DR1 | Natronomonas pharaonis DSM 2160 | 93.33 | 30 | 7.00E-08 | 44.1 |
| G82DR1 | Natronomonas pharaonis DSM 2160 | 93.33 | 30 | 7.00E-08 | 44.1 |
| G82DR1 | Haloferax volcanii DS2 | 93.33 | 30 | 7.00E-08 | 44.1 |
| G88DR1 | Natrialba magadii ATCC 43099 | 93.33 | 30 | 6.00E-08 | 44.1 |
| G88DR1 | Halomicrobium mukohataei DSM 12286 | 100 | 20 | 1.00E-06 | 40.1 |
| G88DR1 | Halomicrobium mukohataei DSM 12286 | 100 | 20 | 1.00E-06 | 40.1 |
| G110DR1 | Saccharomonospora viridis DSM 43017 | 95.83 | 24 | 1.00E-06 | 40.1 |
| G110DR1 | Rhodospirillum rubrum ATCC 11170 | 95.83 | 24 | 1.00E-06 | 40.1 |
| G110DR1 | Rhodospirillum rubrum F11 | 95.83 | 24 | 1.00E-06 | 40.1 |
| G121DR1 | Natrinema sp. J7-2 | 96.67 | 30 | 3.00E-10 | 52 |
| G121DR1 | Halogeometricum borinquense DSM 11551 | 96.67 | 30 | 3.00E-10 | 52 |
| G121DR1 | Haloarcula marismortui ATCC 43049 | 96.67 | 30 | 3.00E-10 | 52 |
| G121DR1 | Haloarcula marismortui ATCC 43049 | 100 | 26 | 3.00E-10 | 52 |
| G121DR1 | Haloferax mediterranei ATCC 33500 | 93.33 | 30 | 7.00E-08 | 44.1 |
| G121DR1 | Halophilic archaeon DL31 | 96.15 | 26 | 7.00E-08 | 44.1 |
| G121DR1 | Halomicrobium mukohataei DSM 12286 | 96.15 | 26 | 7.00E-08 | 44.1 |
| G121DR1 | Halomicrobium mukohataei DSM 12286 | 96.15 | 26 | 7.00E-08 | 44.1 |
| G121DR1 | Halorhabdus tiamatea SARL4B | 96.15 | 26 | 7.00E-08 | 44.1 |
| G121DR1 | Halorhabdus utahensis DSM 12940 | 96.15 | 26 | 7.00E-08 | 44.1 |
| G121DR1 | Natronomonas pharaonis DSM 2160 | 96.15 | 26 | 7.00E-08 | 44.1 |
| G121DR1 | Natronomonas pharaonis DSM 2160 | 96.15 | 26 | 7.00E-08 | 44.1 |
| G121DR1 | Haloferax volcanii DS2 | 96.15 | 26 | 7.00E-08 | 44.1 |
| G467DR1 | Haloquadratum walsbyi C23 | 100 | 30 | 1.00E-12 | 60 |
| G467DR1 | Haloquadratum walsbyi C23 | 96.67 | 30 | 3.00E-10 | 52 |
| G467DR1 | Haloquadratum walsbyi DSM 16790 | 96 | 25 | 3.00E-07 | 42.1 |
