# Supplementary material for: Determining virus-host interactions and glycerol metabolism profiles in geographically diverse solar salterns with metagenomics
Source: PeerJ. 2017 Jan 10;5:e2844. doi: 10.7717/peerj.2844 (PMC5228507; doi:10.7717/peerj.2844)
Supplement: Table S4 [file peerj-05-2844-s011.docx]

Table S4: Query sequences used for reference-guided CRISPR search and associated archaeal hosts

| Pattern (Query) Number | Query DR Taxonomic Affiliation | Query Sequence |
| --- | --- | --- |
| 0 | Haloferax volcanii DS2 | GTTTCAGACGAACCCTTGTGGGGTTGAAGC |
| 1 | Haloquadratum walsbyi DSM 16790 | GTTTCAGATGAACCCTTGATGGGTT |
| 2 | Halorubrum lacusprofundi ATCC 49239 | TCGCCGTTCTCCTCTTCGCCGTTCTC |
| 3 | Halorubrum lacusprofundi ATCC 49239 | GCTTCAAACCCACGAGGGGTTCGGCTGAAAG |
| 4 | Halorubrum lacusprofundi ATCC 49239 | ACTACCCCACTATTTCCGCTGTTAG |
| 5 | Halorubrum lacusprofundi ATCC 49239 | GTCGAGACACTCAGAAAACCCAGCACGGGATTGAAAC |
| 6 | Haloferax volcanii DS2 | GTTTCAGACGAACCCTTGTGGGATTGAAGC |
| 7 | Haloferax volcanii DS2 | GGTTTCAGACGAACCCTTGTGGGTTTGAAGC |
| 8 | Halogeometricum borinquense DSM 11551 | GCTTCAACCCCACAAGGGTTCGTCTGTAAC |
| 9 | Haloquadratum walsbyi C23 | GTTGCAACGAAGAGAAAACCCGCTAAGGGATTGAAAC |
| 10 | Haloquadratum walsbyi C23 | GTTTCAGATGAACCCTTGTTGGGTTGAAGT |
| 11 | Haloquadratum walsbyi C23 | GTTTCAGATGAACCCTTGATGGGTTGAAGT |
| 12 | Haloferax mediterranei ATCC 33500 | GGTTACAGACGGACCCTCGTTGGGTTGAAG |
| 13 | Haloferax mediterranei ATCC 33500 | GTTACAGACGAACCCTAGTTGGGTTGAAGC |
| 14 | Haloferax mediterranei ATCC 33500 | GTTACAGACGAACCCTAGTTGGGTTGAAGC |
| 15 | Haloferax mediterranei ATCC 33500 | GCTTCAACCCAATTAGGGTTCGTCTGTAAC |
| 16 | Haloarcula marismortui ATCC 43049 | GCTTCAACCCCACAAGGGTCCGTCTGAAAC |
| 17 | Haloarcula marismortui ATCC 43049 | GTTACAGACGGACCCTCGTGGGGTTGAAGC |
| 18 | Haloarcula marismortui ATCC 43049 | GTTTCAGACGGACCCTTGGGCGGTTGAAGT |
| 19 | Natronomonas pharaonis DSM 2160 | GTTTCAGACGAACCCTTGTGGGGTTGAAGC |
| 20 | Natronomonas pharaonis DSM 2160 | GTTTCAGACGAACCCTTGTGGGGTTGAAGC |
| 21 | Natronomonas pharaonis DSM 2160 | GTCGAGACGGACTGAAAACCCAGAACGGGATTGAAAC |
| 22 | Natronomonas pharaonis DSM 2160 | GTCGAGACGGACTGAAAACCCAGAACGGGATTGAAAC |
| 23 | Halorhabdus utahensis DSM 12940 | GCTTCAACCCCACGAGGGTCCGTCTGAAAC |
| 24 | Halorhabdus tiamatea SARL4B | GCTTCAACCCCACGAGGGTCCGTCTGAAAC |
| 25 | Halomicrobium mukohataei DSM 12286 | GCTTCAATCCCACAAGGGTCCGTCTGAAAC |
| 26 | Halomicrobium mukohataei DSM 12286 | GCTTCAATCCCACAAGGGTCCGTCTGAAAC |
| 27 | Haloarcula hispanica N601 | GCTTCAACCCCACGAGGGTTCGTCTGAAAC |
| 28 | Haloarcula hispanica ATCC 33960 | GCTTCAACCCCACGAGGGTTCGTCTGAAAC |
