# Supplementary material for: Determining virus-host interactions and glycerol metabolism profiles in geographically diverse solar salterns with metagenomics
Source: PeerJ. 2017 Jan 10;5:e2844. doi: 10.7717/peerj.2844 (PMC5228507; doi:10.7717/peerj.2844)
Supplement: Table S5 — CRISPR spacers were detected with the reference-guided method. [file peerj-05-2844-s012.docx]

Table S5: Summary of CRISPR virus-host pairings in Cahuil (C34) metagenome; CRISPR spacers were detected with the reference-guided method

| Putative host | Matched virus |
| --- | --- |
| Haloquadratum walsbyi | CVcontig00192 |
| Haloquadratum walsbyi | LTVcontig998975 |
| Haloquadratum walsbyi | eHP-11 |
| Haloquadratum walsbyi | CVcontig00213 |
| Halorhabdus utahensis (or Haloarcula hispanica) | eHP-31 |
| Haloarcula marismortui | CVcontig00192 |
| Haloarcula marismortui | eHP-11 |
