# Supplementary material for: Determining virus-host interactions and glycerol metabolism profiles in geographically diverse solar salterns with metagenomics
Source: PeerJ. 2017 Jan 10;5:e2844. doi: 10.7717/peerj.2844 (PMC5228507; doi:10.7717/peerj.2844)
Supplement: Table S6 — CRISPR spacers were detected with the reference-guided method. [file peerj-05-2844-s013.docx]

Table S6: Summary of CRISPR virus-host pairings in SS13 metagenome; CRISPR spacers were detected with the reference-guided method

| Putative host | Matched virus |
| --- | --- |
| Haloquadratum walsbyi | CVcontig00305 |
