# Supplementary material for: Determining virus-host interactions and glycerol metabolism profiles in geographically diverse solar salterns with metagenomics
Source: PeerJ. 2017 Jan 10;5:e2844. doi: 10.7717/peerj.2844 (PMC5228507; doi:10.7717/peerj.2844)
Supplement: Table S10 — CRISPR spacers were detected with the reference-guided method. [file peerj-05-2844-s017.docx]

Table S10: Summary of CRISPR virus-host pairings in SS37 metagenome; CRISPR spacers were detected with the reference-guided method

| Putative host | Matched virus |
| --- | --- |
| Haloquadratum walsbyi | eHP-2 |
| Haloquadratum walsbyi | eHP-42 |
| Haloquadratum walsbyi | eHP-5 |
| Haloquadratum walsbyi | eHP-29 |
| Haloquadratum walsbyi | eHP-E5 |
| Haloquadratum walsbyi | eHP-22 |
| Haloquadratum walsbyi | eHP-24 |
| Haloquadratum walsbyi | eHP-37 |
| Haloquadratum walsbyi | eHP-38 |
| Haloquadratum walsbyi | eHP-39 |
| Haloquadratum walsbyi | eHP-41 |
| Haloquadratum walsbyi | eHP-D7 |
| Halorhabdus utahensis (or Haloarcula hispanica) | CVcontig00024 |
| Halorhabdus utahensis (or Haloarcula hispanica) | eHP-37 |
| Halorhabdus utahensis (or Haloarcula hispanica) | eHP-38 |
| Halorhabdus utahensis (or Haloarcula hispanica) | eHP-41 |
| Halorhabdus utahensis (or Haloarcula hispanica) | eHP-42 |
| Halorhabdus utahensis (or Haloarcula hispanica) | eHP-D7 |
| Halorhabdus utahensis (or Haloarcula hispanica) | eHP-E5 |
