# Supplementary material for: Determining virus-host interactions and glycerol metabolism profiles in geographically diverse solar salterns with metagenomics
Source: PeerJ. 2017 Jan 10;5:e2844. doi: 10.7717/peerj.2844 (PMC5228507; doi:10.7717/peerj.2844)
Supplement: Table S11 — The library was aligned against Newbler contigs assembled from the Cahuil/C34, combined Chula Vista, and combined Santa Pola/Isla Cristina metagenomes. [file peerj-05-2844-s018.docx]

Table S11: The library of *cas* genes identified in Halobacteriales genomes that were aligned against Newbler contigs assembled from the Cahuil/C34, combined Chula Vista, and combined Santa Pola/Isla Cristina metagenomes

| GI | GB | Description |
| --- | --- | --- |
| 749702013 | AJF26807.1 | CRISPR-associated protein [Haloarcula sp. CBA1115] |
| 445788732 | EMA39438.1 | CRISPR-associated protein, partial [Halococcus morrhuae DSM 1307] |
| 320548909 | EFW90576.1 | CRISPR-associated protein [Haladaptatus paucihalophilus DX253] |
| 55228999 | AAV44422.1 | CRISPR-associated protein (plasmid) [Haloarcula marismortui ATCC 43049] |
| 491183000 | WP_005041362.1 | crispr-associated protein [Halococcus salifodinae] |
| 445804137 | EMA54400.1 | crispr-associated protein [Halococcus salifodinae DSM 8989] |
| 910232731 | WP_050037906.1 | CRISPR-associated protein [Haloarcula sp. CBA1115] |
| 910017809 | WP_049972516.1 | CRISPR-associated protein [Haladaptatus cibarius] |
| 257171180 | ACV48939.1 | CRISPR-associated protein, Csh2 family [Halomicrobium mukohataei DSM 12286] |
| 256691292 | ACV11629.1 | CRISPR-associated protein, Csh2 family [Halorhabdus utahensis DSM 12940] |
| 257171185 | ACV48944.1 | CRISPR-associated protein Cas2 [Halomicrobium mukohataei DSM 12286] |
| 257171184 | ACV48943.1 | CRISPR-associated protein Cas1 [Halomicrobium mukohataei DSM 12286] |
| 257171181 | ACV48940.1 | CRISPR-associated protein Cas5, Hmari subtype [Halomicrobium mukohataei DSM 12286] |
| 256691291 | ACV11628.1 | CRISPR-associated protein Cas5, Hmari subtype [Halorhabdus utahensis DSM 12940] |
| 256691288 | ACV11625.1 | CRISPR-associated protein Cas1 [Halorhabdus utahensis DSM 12940] |
| 256691287 | ACV11624.1 | CRISPR-associated protein Cas2 [Halorhabdus utahensis DSM 12940] |
| 257171183 | ACV48942.1 | CRISPR-associated protein Cas4 [Halomicrobium mukohataei DSM 12286] |
| 256691289 | ACV11626.1 | CRISPR-associated protein Cas4 [Halorhabdus utahensis DSM 12940] |
| 256691294 | ACV11631.1 | CRISPR-associated protein Cas6 [Halorhabdus utahensis DSM 12940] |
| 257171179 | ACV48938.1 | CRISPR-associated protein, Csh1 family [Halomicrobium mukohataei DSM 12286] |
| 257171178 | ACV48937.1 | CRISPR-associated protein Cas6 [Halomicrobium mukohataei DSM 12286] |
| 256691293 | ACV11630.1 | CRISPR-associated protein, Csh1 family [Halorhabdus utahensis DSM 12940] |
| 497448090 | WP_009762288.1 | CRISPR-associated HD domain protein [Halobacterium sp. DL1] |
| 497448087 | WP_009762285.1 | CRISPR-associated protein Csh1 domain protein [Halobacterium sp. DL1] |
| 543101072 | ERJ06791.1 | CRISPR-associated protein Csh2 family protein [Halorhabdus tiamatea SARL4B] |
| 543101071 | ERJ06790.1 | CRISPR-associated protein Csh1 family protein [Halorhabdus tiamatea SARL4B] |
| 499642613 | WP_011323347.1 | CRISPR-associated protein Cas3 [Natronomonas pharaonis] |
| 445756790 | EMA08148.1 | CRISPR-associated protein Cas6 [Haloarcula sinaiiensis ATCC 33800] |
| 749702016 | AJF26810.1 | CRISPR-associated protein Cas2 [Haloarcula sp. CBA1115] |
| 749702015 | AJF26809.1 | CRISPR-associated protein Cas1 [Haloarcula sp. CBA1115] |
| 749702014 | AJF26808.1 | CRISPR-associated protein Cas4 [Haloarcula sp. CBA1115] |
| 749702011 | AJF26805.1 | CRISPR-associated protein Csc2 [Haloarcula sp. CBA1115] |
| 749702010 | AJF26804.1 | CRISPR-associated protein Cas6 [Haloarcula sp. CBA1115] |
| 573487046 | AHG05451.1 | CRISPR-associated protein Cas2 (plasmid) [Halobacterium sp. DL1] |
| 573487045 | AHG05450.1 | CRISPR-associated protein Cas1 (plasmid) [Halobacterium sp. DL1] |
| 573487042 | AHG05447.1 | CRISPR-associated protein Cas5 (plasmid) [Halobacterium sp. DL1] |
| 573487041 | AHG05446.1 | CRISPR-associated protein Csh2 (plasmid) [Halobacterium sp. DL1] |
| 573487040 | AHG05445.1 | CRISPR-associated protein Csh1 (plasmid) [Halobacterium sp. DL1] |
| 573487039 | AHG05444.1 | CRISPR-associated protein Cas6 (plasmid) [Halobacterium sp. DL1] |
| 491106984 | WP_004966752.1 | CRISPR-associated protein Cas6 [Haloarcula sinaiiensis] |
| 573487044 | AHG05449.1 | CRISPR-associated protein Cas4 (plasmid) [Halobacterium sp. DL1] |
| 556695528 | ESP86785.1 | CRISPR-associated protein Cas2 [Candidatus Halobonum tyrrellensis G22] |
| 556695527 | ESP86784.1 | CRISPR-associated protein Cas1 [Candidatus Halobonum tyrrellensis G22] |
| 556695524 | ESP86781.1 | CRISPR-associated protein Cas5, Hmari subtype [Candidatus Halobonum tyrrellensis G22] |
| 556695523 | ESP86780.1 | CRISPR-associated protein Cas7/Csh2, subtype I-B/HMARI [Candidatus Halobonum tyrrellensis G22] |
| 445806733 | EMA56844.1 | CRISPR-associated protein Cas5 [Halococcus thailandensis JCM 13552] |
| 445806731 | EMA56842.1 | Csh2 family CRISPR-associated protein [Halococcus thailandensis JCM 13552] |
| 445806729 | EMA56840.1 | CRISPR-associated protein Cas6 [Halococcus thailandensis JCM 13552] |
| 445806440 | EMA56569.1 | CRISPR-associated Cas1 family protein [Halococcus thailandensis JCM 13552] |
| 445806439 | EMA56568.1 | CRISPR-associated protein Cas2 [Halococcus thailandensis JCM 13552] |
| 445804135 | EMA54398.1 | CRISPR-associated Cas5h family protein [Halococcus salifodinae DSM 8989] |
| 445788730 | EMA39436.1 | CRISPR-associated protein Cas6 [Halococcus morrhuae DSM 1307] |
| 445774126 | EMA25148.1 | CRISPR-associated protein Cas6 [Haloarcula argentinensis DSM 12282] |
| 445774124 | EMA25146.1 | CRISPR-associated protein, Csh2 family [Haloarcula argentinensis DSM 12282] |
| 445774123 | EMA25145.1 | CRISPR-associated protein Cas5, Hmari subtype [Haloarcula argentinensis DSM 12282] |
| 445774120 | EMA25142.1 | CRISPR-associated protein Cas1 [Haloarcula argentinensis DSM 12282] |
| 445774119 | EMA25141.1 | CRISPR-associated protein Cas2 [Haloarcula argentinensis DSM 12282] |
| 445767708 | EMA18802.1 | Csh2 family CRISPR-associated protein [Haloarcula californiae ATCC 33799] |
| 445767707 | EMA18801.1 | CRISPR-associated protein Cas5, Hmari subtype [Haloarcula californiae ATCC 33799] |
| 445756797 | EMA08155.1 | CRISPR-associated protein Cas2 [Haloarcula sinaiiensis ATCC 33800] |
| 445756796 | EMA08154.1 | CRISPR-associated protein Cas1 [Haloarcula sinaiiensis ATCC 33800] |
| 445756792 | EMA08150.1 | CRISPR-associated protein, Csh2 family [Haloarcula sinaiiensis ATCC 33800] |
| 320548914 | EFW90581.1 | CRISPR-associated protein Cas2 [Haladaptatus paucihalophilus DX253] |
| 320548913 | EFW90580.1 | CRISPR-associated protein Cas1 [Haladaptatus paucihalophilus DX253] |
| 320548907 | EFW90574.1 | CRISPR-associated protein Cas6 [Haladaptatus paucihalophilus DX253] |
| 497448092 | WP_009762290.1 | CRISPR-associated protein Cas2 [Halobacterium sp. DL1] |
| 497448091 | WP_009762289.1 | CRISPR-associated protein Cas1 [Halobacterium sp. DL1] |
| 497448089 | WP_009762287.1 | CRISPR-associated protein Cas5 [Halobacterium sp. DL1] |
| 497448088 | WP_009762286.1 | CRISPR-associated protein Csh2 [Halobacterium sp. DL1] |
| 494345545 | WP_007188931.1 | CRISPR-associated protein Cas5 [Haloarcula californiae] |
| 491677582 | WP_005533724.1 | CRISPR-associated protein Cas5 [Haloarcula argentinensis] |
| 491182996 | WP_005041358.1 | CRISPR-associated protein Cas5 [Halococcus salifodinae] |
| 543416120 | GAD52992.1 | CRISPR-associated protein, Cas5h family [Halarchaeum acidiphilum MH1-52-1] |
| 543414359 | GAD53621.1 | CRISPR-associated protein Csc2 [Halarchaeum acidiphilum MH1-52-1] |
| 543414355 | GAD53617.1 | CRISPR-associated protein Cas1 [Halarchaeum acidiphilum MH1-52-1] |
| 543414354 | GAD53616.1 | CRISPR-associated protein Cas2 [Halarchaeum acidiphilum MH1-52-1] |
| 543413946 | GAD53845.1 | CRISPR-associated protein Cas1 [Halarchaeum acidiphilum MH1-52-1] |
| 543413945 | GAD53844.1 | CRISPR-associated protein Cas2 [Halarchaeum acidiphilum MH1-52-1] |
| 769188913 | WP_044957276.1 | CRISPR-associated protein Cas2 [Halarchaeum acidiphilum] |
| 769188805 | WP_044957195.1 | CRISPR-associated protein Cas2 [Halarchaeum acidiphilum] |
| 769188798 | WP_044957190.1 | CRISPR-associated protein Cas6 [Halarchaeum acidiphilum] |
| 769182643 | WP_044952751.1 | CRISPR-associated protein Csh1 [Haloarcula hispanica] |
| 769182640 | WP_044952748.1 | CRISPR-associated protein Cas6 [Haloarcula hispanica] |
| 746356220 | WP_039400886.1 | CRISPR-associated protein Csh1 [haloarchaeon 3A1_DGR] |
| 746356216 | WP_039400882.1 | CRISPR-associated protein Cas6 [haloarchaeon 3A1_DGR] |
| 76558138 | CAI49725.1 | CRISPR-associated protein Csc1 [Natronomonas pharaonis DSM 2160] |
| 76558137 | CAI49724.1 | CRISPR-associated protein Cas7 [Natronomonas pharaonis DSM 2160] |
| 76558136 | CAI49723.1 | CRISPR-associated protein Cas10d [Natronomonas pharaonis DSM 2160] |
| 76558134 | CAI49721.1 | CRISPR-associated DNA-binding protein Csm6 [Natronomonas pharaonis DSM 2160] |
| 648486250 | WP_026178001.1 | CRISPR-associated protein Csh1 [Haladaptatus paucihalophilus] |
| 648486249 | WP_026178000.1 | CRISPR-associated protein Cas5 [Haladaptatus paucihalophilus] |
| 648486247 | WP_026177998.1 | CRISPR-associated protein Cas1 [Haladaptatus paucihalophilus] |
| 528526430 | CCQ33719.1 | CRISPR-associated protein, Cas2 [Halorhabdus tiamatea SARL4B] |
| 528526429 | CCQ33718.1 | CRISPR-associated protein, Cas1 [Halorhabdus tiamatea SARL4B] |
| 528526426 | CCQ33715.1 | CRISPR-associated protein, Cas5h [Halorhabdus tiamatea SARL4B] |
| 528526425 | CCQ33714.1 | CRISPR-associated protein, Csh2 [Halorhabdus tiamatea SARL4B] |
| 528526424 | CCQ33713.1 | CRISPR-associated protein, Csh1 [Halorhabdus tiamatea SARL4B] |
| 528526423 | CCQ33712.1 | CRISPR-associated protein, Cas6 [Halorhabdus tiamatea SARL4B] |
| 564124632 | AHB67952.1 | CRISPR-associated protein Cas2 (plasmid) [Haloarcula hispanica N601] |
| 564124631 | AHB67951.1 | CRISPR-associated protein Cas1 (plasmid) [Haloarcula hispanica N601] |
| 564124628 | AHB67948.1 | CRISPR-associated protein Cas5 (plasmid) [Haloarcula hispanica N601] |
| 564124627 | AHB67947.1 | CRISPR-associated protein Csh2 (plasmid) [Haloarcula hispanica N601] |
| 564124626 | AHB67946.1 | CRISPR-associated protein Csh1 (plasmid) [Haloarcula hispanica N601] |
| 564124625 | AHB67945.1 | CRISPR-associated protein Cas6 (plasmid) [Haloarcula hispanica N601] |
| 543101077 | ERJ06796.1 | CRISPR-associated endoribonuclease Cas2 2 protein [Halorhabdus tiamatea SARL4B] |
| 543101076 | ERJ06795.1 | CRISPR-associated endonuclease Cas1 2 protein [Halorhabdus tiamatea SARL4B] |
| 543101074 | ERJ06793.1 | CRISPR-associated helicase Cas3 family protein [Halorhabdus tiamatea SARL4B] |
| 543101073 | ERJ06792.1 | CRISPR-associated protein Cas5 Hmari subtype [Halorhabdus tiamatea SARL4B] |
| 543101070 | ERJ06789.1 | CRISPR-associated protein Cas6 [Halorhabdus tiamatea SARL4B] |
| 55229004 | AAV44427.1 | CRISPR-associated protein Cas2 (plasmid) [Haloarcula marismortui ATCC 43049] |
| 55229003 | AAV44426.1 | CRISPR-associated protein Cas1 (plasmid) [Haloarcula marismortui ATCC 43049] |
| 343784867 | AEM58843.1 | CRISPR-associated protein Cas6 [Haloarcula hispanica ATCC 33960] |
| 343784866 | AEM58842.1 | CRISPR-associated protein, Csh1 family [Haloarcula hispanica ATCC 33960] |
| 343784865 | AEM58841.1 | CRISPR-associated protein, Csh2 family [Haloarcula hispanica ATCC 33960] |
| 343784864 | AEM58840.1 | CRISPR-associated protein Cas5, Hmari subtype [Haloarcula hispanica ATCC 33960] |
| 343784861 | AEM58837.1 | CRISPR-associated protein Cas1 [Haloarcula hispanica ATCC 33960] |
| 343784860 | AEM58836.1 | CRISPR-associated protein Cas2 [Haloarcula hispanica ATCC 33960] |
| 557375040 | WP_023396045.1 | CRISPR-associated protein Cas1 [Candidatus Halobonum tyrrellensis] |
| 557375036 | WP_023396041.1 | CRISPR-associated protein Cas7/Csh2, subtype I-B/HMARI [Candidatus Halobonum tyrrellensis] |
| 557375035 | WP_023396040.1 | CRISPR-associated protein, Csh1 family [Candidatus Halobonum tyrrellensis] |
| 556695522 | ESP86779.1 | CRISPR-associated protein, Csh1 family [Candidatus Halobonum tyrrellensis G22] |
| 545903686 | WP_021780686.1 | CRISPR-associated protein Cas1 [Halarchaeum acidiphilum] |
| 544640163 | WP_021074704.1 | CRISPR-associated protein Csh2 [haloarchaeon 3A1_DGR] |
| 544640159 | WP_021074700.1 | CRISPR-associated protein Cas1 [haloarchaeon 3A1_DGR] |
| 541202045 | ERH10110.1 | CRISPR-associated protein Cas5, Hmari subtype [halophilic archaeon J07HX64] |
| 491106992 | WP_004966758.1 | CRISPR-associated protein Cas1 [Haloarcula sinaiiensis] |
| 491106986 | WP_004966754.1 | CRISPR-associated protein, Csh2 family [Haloarcula sinaiiensis] |
| 519065063 | WP_020220938.1 | CRISPR-associated protein Cas1 [Halarchaeum acidiphilum] |
| 519065059 | WP_020220934.1 | CRISPR-associated protein Csh2 [Halarchaeum acidiphilum] |
| 517069344 | WP_018258162.1 | CRISPR-associated protein Cas6 [Halomicrobium katesii] |
| 517069343 | WP_018258161.1 | CRISPR-associated protein Csh1 [Halomicrobium katesii] |
| 517069342 | WP_018258160.1 | CRISPR-associated protein Csh2 [Halomicrobium katesii] |
| 517069341 | WP_018258159.1 | CRISPR-associated protein Cas5 [Halomicrobium katesii] |
| 517069338 | WP_018258156.1 | CRISPR-associated protein Cas1 [Halomicrobium katesii] |
| 495010973 | WP_007736985.1 | CRISPR-associated protein Csc2 [Halococcus thailandensis] |
| 495010970 | WP_007736982.1 | CRISPR-associated protein Cas4 [Halococcus thailandensis] |
| 495010465 | WP_007736477.1 | CRISPR-associated protein Cas5 [Halococcus thailandensis] |
| 495010461 | WP_007736473.1 | CRISPR-associated protein, Csh1 family [Halococcus thailandensis] |
| 494345549 | WP_007188933.1 | CRISPR-associated Csh1 family protein [Haloarcula californiae] |
| 491677585 | WP_005533727.1 | CRISPR-associated protein Cas6 [Haloarcula argentinensis] |
| 491677584 | WP_005533726.1 | CRISPR-associated protein, Csh1 family [Haloarcula argentinensis] |
| 491677583 | WP_005533725.1 | CRISPR-associated protein, Csh2 family [Haloarcula argentinensis] |
| 491677579 | WP_005533721.1 | CRISPR-associated protein Cas1 [Haloarcula argentinensis] |
| 503796723 | WP_014030717.1 | CRISPR-associated protein Csh2 [Haloarcula hispanica] |
| 503796722 | WP_014030716.1 | CRISPR-associated protein Cas5 [Haloarcula hispanica] |
| 495800278 | WP_008524857.1 | CRISPR-associated protein Cas6 [Halorhabdus tiamatea] |
| 495800276 | WP_008524855.1 | CRISPR-associated protein Csh1 [Halorhabdus tiamatea] |
| 495800274 | WP_008524853.1 | CRISPR-associated protein Csh2 [Halorhabdus tiamatea] |
| 495800273 | WP_008524852.1 | CRISPR-associated protein Cas5 [Halorhabdus tiamatea] |
| 495800270 | WP_008524849.1 | CRISPR-associated protein Cas1 [Halorhabdus tiamatea] |
| 495257574 | WP_007982329.1 | CRISPR-associated protein Csh2 [Haladaptatus paucihalophilus] |
| 506269429 | WP_015789204.1 | CRISPR-associated protein Csh1 [Halorhabdus utahensis] |
| 506269428 | WP_015789203.1 | CRISPR-associated protein Csh2 [Halorhabdus utahensis] |
| 506269427 | WP_015789202.1 | CRISPR-associated protein Cas5 [Halorhabdus utahensis] |
| 506244006 | WP_015763781.1 | CRISPR-associated protein Csh2 [Halomicrobium mukohataei] |
| 506244005 | WP_015763780.1 | CRISPR-associated protein Csh1 [Halomicrobium mukohataei] |
| 499642615 | WP_011323349.1 | CRISPR-associated protein Cas1 [Natronomonas pharaonis] |
| 499642614 | WP_011323348.1 | CRISPR-associated protein Cas4 [Natronomonas pharaonis] |
| 499642612 | WP_011323346.1 | CRISPR-associated protein Csc1 [Natronomonas pharaonis] |
| 499642611 | WP_011323345.1 | CRISPR-associated protein Csc2 [Natronomonas pharaonis] |
| 499642610 | WP_011323344.1 | CRISPR-associated protein Csc3 [Natronomonas pharaonis] |
| 499642609 | WP_011323343.1 | CRISPR-associated protein Cas6 [Natronomonas pharaonis] |
| 499541488 | WP_011222271.1 | CRISPR-associated protein Cas1 [Haloarcula marismortui] |
| 499541485 | WP_011222268.1 | CRISPR-associated protein Cas5 [Haloarcula marismortui] |
| 499541484 | WP_011222267.1 | CRISPR-associated protein Csh2 [Haloarcula marismortui] |
| 499541483 | WP_011222266.1 | CRISPR-associated protein Csh1 [Haloarcula marismortui] |
| 499541482 | WP_011222265.1 | CRISPR-associated protein Cas6 [Haloarcula marismortui] |
| 506269430 | WP_015789205.1 | CRISPR-associated protein Cas6 [Halorhabdus utahensis] |
| 506269424 | WP_015789199.1 | CRISPR-associated protein Cas1 [Halorhabdus utahensis] |
| 506244010 | WP_015763785.1 | CRISPR-associated protein Cas1 [Halomicrobium mukohataei] |
| 506244007 | WP_015763782.1 | CRISPR-associated protein Cas5 [Halomicrobium mukohataei] |
| 506244004 | WP_015763779.1 | CRISPR-associated protein Cas6 [Halomicrobium mukohataei] |
| 503796719 | WP_014030713.1 | CRISPR-associated protein Cas1 [Haloarcula hispanica] |
| 445806730 | EMA56841.1 | CRISPR-associated protein, Csh1 family [Halococcus thailandensis JCM 13552] |
| 445806444 | EMA56573.1 | CRISPR-associated protein Csc2 [Halococcus thailandensis JCM 13552] |
| 445806441 | EMA56570.1 | CRISPR-associated protein Cas4 [Halococcus thailandensis JCM 13552] |
| 445804136 | EMA54399.1 | CRISPR-associated protein Csh2 [Halococcus salifodinae DSM 8989] |
| 445774125 | EMA25147.1 | CRISPR-associated protein, Csh1 family [Haloarcula argentinensis DSM 12282] |
| 445767709 | EMA18803.1 | CRISPR-associated Csh1 family protein [Haloarcula californiae ATCC 33799] |
| 445767704 | EMA18798.1 | CRISPR-associated Cas2 family protein [Haloarcula californiae ATCC 33799] |
| 445756793 | EMA08151.1 | CRISPR-associated protein Cas5, Hmari subtype [Haloarcula sinaiiensis ATCC 33800] |
| 320548910 | EFW90577.1 | CRISPR-associated protein Cas5, Hmari subtype [Haladaptatus paucihalophilus DX253] |
| 910243469 | WP_050048135.1 | CRISPR-associated protein Cas2 [Halanaeroarchaeum sulfurireducens] |
| 910243468 | WP_050048134.1 | CRISPR-associated protein Cas1 [Halanaeroarchaeum sulfurireducens] |
| 910243467 | WP_050048133.1 | CRISPR-associated protein Cas4 [Halanaeroarchaeum sulfurireducens] |
| 910243465 | WP_050048131.1 | CRISPR-associated protein Cas5 [Halanaeroarchaeum sulfurireducens] |
| 910243464 | WP_050048130.1 | CRISPR-associated protein Csh2 [Halanaeroarchaeum sulfurireducens] |
| 910243463 | WP_050048129.1 | CRISPR-associated protein Csh1 [Halanaeroarchaeum sulfurireducens] |
| 910243462 | WP_050048128.1 | CRISPR-associated protein Cas6 [Halanaeroarchaeum sulfurireducens] |
| 910232734 | WP_050037909.1 | CRISPR-associated protein Cas2 [Haloarcula sp. CBA1115] |
| 910232733 | WP_050037908.1 | CRISPR-associated protein Cas1 [Haloarcula sp. CBA1115] |
| 910232732 | WP_050037907.1 | CRISPR-associated protein Cas4 [Haloarcula sp. CBA1115] |
| 910232729 | WP_050037904.1 | CRISPR-associated protein Csc2 [Haloarcula sp. CBA1115] |
| 910232728 | WP_050037903.1 | CRISPR-associated protein Cas6 [Haloarcula sp. CBA1115] |
| 497448086 | WP_009762284.1 | CRISPR-associated protein Cas6 [Halobacterium sp. DL1] |
| 910017932 | WP_049972639.1 | CRISPR-associated protein Csh1 [Haladaptatus cibarius] |
| 910017856 | WP_049972563.1 | CRISPR-associated protein Cas2 [Haladaptatus cibarius] |
| 910017855 | WP_049972562.1 | CRISPR-associated protein Cas1 [Haladaptatus cibarius] |
| 910017854 | WP_049972561.1 | CRISPR-associated protein Cas4 [Haladaptatus cibarius] |
| 910017852 | WP_049972559.1 | CRISPR-associated protein Cas5 [Haladaptatus cibarius] |
| 910017851 | WP_049972558.1 | CRISPR-associated protein Csh2 [Haladaptatus cibarius] |
| 910017850 | WP_049972557.1 | CRISPR-associated protein Cas6 [Haladaptatus cibarius] |
| 910017813 | WP_049972520.1 | CRISPR-associated protein Cas6 [Haladaptatus cibarius] |
| 910017811 | WP_049972518.1 | CRISPR-associated protein Csc2 [Haladaptatus cibarius] |
| 910017808 | WP_049972515.1 | CRISPR-associated protein Cas4 [Haladaptatus cibarius] |
| 910017807 | WP_049972514.1 | CRISPR-associated protein Cas1 [Haladaptatus cibarius] |
| 910017806 | WP_049972513.1 | CRISPR-associated protein Cas2 [Haladaptatus cibarius] |
| 909704826 | WP_049948027.1 | CRISPR-associated protein Cas4 [Candidatus Halobonum tyrrellensis] |
| 909704824 | WP_049948025.1 | CRISPR-associated protein Cas5 [Candidatus Halobonum tyrrellensis] |
| 909704823 | WP_049948024.1 | CRISPR-associated protein Cas6 [Candidatus Halobonum tyrrellensis] |
| 909701387 | WP_049944624.1 | CRISPR-associated protein Cas2 [Haloarcula californiae] |
| 909697750 | WP_049940987.1 | CRISPR-associated protein Csh1 [Haladaptatus paucihalophilus] |
| 909696352 | WP_049939589.1 | CRISPR-associated protein Cas2 [Natronomonas pharaonis] |
| 909676135 | WP_049919489.1 | CRISPR-associated protein Cas4 [Haloarcula sinaiiensis] |
| 909676134 | WP_049919488.1 | CRISPR-associated protein Cas5 [Haloarcula sinaiiensis] |
| 909673035 | WP_049916389.1 | CRISPR-associated protein Cas6 [Halococcus morrhuae] |
| 909669322 | WP_049912676.1 | CRISPR-associated protein Cas1 [Halococcus thailandensis] |
| 909669240 | WP_049912594.1 | CRISPR-associated protein Cas6 [Halococcus thailandensis] |
| 909660037 | WP_049903450.1 | CRISPR-associated protein Cas6 [Halococcus sp. 197A] |
| 909660035 | WP_049903448.1 | CRISPR-associated protein Csh1 [Halococcus sp. 197A] |
| 909660032 | WP_049903445.1 | CRISPR-associated protein Csh2 [Halococcus sp. 197A] |
| 909660029 | WP_049903442.1 | CRISPR-associated protein Cas5 [Halococcus sp. 197A] |
| 909660024 | WP_049903437.1 | CRISPR-associated protein Cas4 [Halococcus sp. 197A] |
| 909660021 | WP_049903434.1 | CRISPR-associated protein Cas1 [Halococcus sp. 197A] |
| 909660019 | WP_049903432.1 | CRISPR-associated protein Cas2 [Halococcus sp. 197A] |
| 908685970 | WP_049817745.1 | CRISPR-associated protein Csh1 [haloarchaeon 3A1_DGR] |
| 822661539 | AKH97363.1 | CRISPR-associated protein Cas2 [Halanaeroarchaeum sulfurireducens] |
| 822661538 | AKH97362.1 | CRISPR-associated protein Cas1 [Halanaeroarchaeum sulfurireducens] |
| 822661537 | AKH97361.1 | CRISPR-associated protein Cas4 [Halanaeroarchaeum sulfurireducens] |
| 822661536 | AKH97360.1 | CRISPR-associated protein Cas3 [Halanaeroarchaeum sulfurireducens] |
| 822661535 | AKH97359.1 | CRISPR-associated protein Cas5 [Halanaeroarchaeum sulfurireducens] |
| 822661534 | AKH97358.1 | CRISPR-associated protein Cas7/Csh2 [Halanaeroarchaeum sulfurireducens] |
| 822661533 | AKH97357.1 | CRISPR-associated protein Cas8b/Csh1 [Halanaeroarchaeum sulfurireducens] |
| 822661532 | AKH97356.1 | CRISPR-associated protein Cas6 [Halanaeroarchaeum sulfurireducens] |
| 769188803 | WP_044957193.1 | CRISPR-associated protein Cas4 [Halarchaeum acidiphilum] |
| 746356226 | WP_039400892.1 | CRISPR-associated protein Cas4 [haloarchaeon 3A1_DGR] |
| 648486248 | WP_026177999.1 | CRISPR-associated protein Cas4 [Haladaptatus paucihalophilus] |
| 528526428 | CCQ33717.1 | CRISPR-associated protein, Cas4a [Halorhabdus tiamatea SARL4B] |
| 564124630 | AHB67950.1 | CRISPR-associated protein Cas4 (plasmid) [Haloarcula hispanica N601] |
| 543101075 | ERJ06794.1 | CRISPR-associated protein Cas4 [Halorhabdus tiamatea SARL4B] |
| 55229002 | AAV44425.1 | CRISPR-associated protein Cas4 (plasmid) [Haloarcula marismortui ATCC 43049] |
| 343784862 | AEM58838.1 | CRISPR-associated protein Cas4 [Haloarcula hispanica ATCC 33960] |
| 556695526 | ESP86783.1 | CRISPR-associated protein Cas4 [Candidatus Halobonum tyrrellensis G22] |
| 517069339 | WP_018258157.1 | CRISPR-associated protein Cas4 [Halomicrobium katesii] |
| 491677580 | WP_005533722.1 | CRISPR-associated protein Cas4 [Haloarcula argentinensis] |
| 491182998 | WP_005041360.1 | CRISPR-associated protein Csh2 [Halococcus salifodinae] |
| 495800271 | WP_008524850.1 | CRISPR-associated protein Cas4 [Halorhabdus tiamatea] |
| 499541487 | WP_011222270.1 | CRISPR-associated protein Cas4 [Haloarcula marismortui] |
| 506269425 | WP_015789200.1 | CRISPR-associated protein Cas4 [Halorhabdus utahensis] |
| 506244009 | WP_015763784.1 | CRISPR-associated protein Cas4 [Halomicrobium mukohataei] |
| 503796720 | WP_014030714.1 | CRISPR-associated protein Cas4 [Haloarcula hispanica] |
| 445774121 | EMA25143.1 | CRISPR-associated protein Cas4 [Haloarcula argentinensis DSM 12282] |
| 445756795 | EMA08153.1 | CRISPR-associated protein Cas4 [Haloarcula sinaiiensis ATCC 33800] |
| 320548912 | EFW90579.1 | CRISPR-associated Cas4 family protein [Haladaptatus paucihalophilus DX253] |
| 543414360 | GAD53622.1 | CRISPR associated protein Csc3 [Halarchaeum acidiphilum MH1-52-1] |
| 257168932 | ACV46691.1 | conserved hypothetical protein [Halomicrobium mukohataei DSM 12286] |
| 76558139 | CAI49726.1 | CRISPR-associated helicase Cas3 [Natronomonas pharaonis DSM 2160] |
| 343784863 | AEM58839.1 | CRISPR-associated helicase Cas3 [Haloarcula hispanica ATCC 33960] |
| 519065061 | WP_020220936.1 | hypothetical protein [Halarchaeum acidiphilum] |
| 516847548 | WP_018129105.1 | hypothetical protein [Haladaptatus paucihalophilus] |
| 493940704 | WP_006884856.1 | hypothetical protein [Halosimplex carlsbadense] |
| 491677581 | WP_005533723.1 | CRISPR-associated helicase Cas3 [Haloarcula argentinensis] |
| 445804138 | EMA54401.1 | hypothetical protein C450_06210 [Halococcus salifodinae DSM 8989] |
| 445774122 | EMA25144.1 | CRISPR-associated helicase Cas3 [Haloarcula argentinensis DSM 12282] |
| 445670557 | ELZ23156.1 | hypothetical protein C475_15944 [Halosimplex carlsbadense 2-9-1] |
| 320549835 | EFW91492.1 | hypothetical protein ZOD2009_14031 [Haladaptatus paucihalophilus DX253] |
| 543414357 | GAD53619.1 | CRISPR-associated helicase Cas3 [Halarchaeum acidiphilum MH1-52-1] |
| 556695525 | ESP86782.1 | CRISPR-associated helicase Cas3 [Candidatus Halobonum tyrrellensis G22] |
| 519065260 | WP_020221135.1 | hypothetical protein [Halarchaeum acidiphilum] |
| 491183003 | WP_005041365.1 | hypothetical protein [Halococcus salifodinae] |
| 529147355 | WP_020936219.1 | CRISPR-associated helicase Cas3 [Halorhabdus tiamatea] |
| 648486246 | WP_026177997.1 | CRISPR-associated endonuclease Cas2 [Haladaptatus paucihalophilus] |
| 557375041 | WP_023396046.1 | CRISPR-associated endonuclease Cas2 [Candidatus Halobonum tyrrellensis] |
| 544640158 | WP_021074699.1 | CRISPR-associated endonuclease Cas2 [haloarchaeon 3A1_DGR] |
| 543935980 | WP_021029443.1 | CRISPR-associated endonuclease Cas2 [Halorhabdus tiamatea] |
| 519065263 | WP_020221138.1 | CRISPR-associated endonuclease Cas2 [Halarchaeum acidiphilum] |
| 506269423 | WP_015789198.1 | CRISPR-associated endonuclease Cas2 [Halorhabdus utahensis] |
| 506244011 | WP_015763786.1 | MULTISPECIES: CRISPR-associated endonuclease Cas2 [Halomicrobium] |
| 503796718 | WP_014030712.1 | CRISPR-associated endonuclease Cas2 [Haloarcula hispanica] |
| 499541489 | WP_011222272.1 | CRISPR-associated endonuclease Cas2 [Haloarcula marismortui] |
| 495010967 | WP_007736979.1 | CRISPR-associated endonuclease Cas2 [Halococcus thailandensis] |
| 491677576 | WP_005533718.1 | CRISPR-associated endonuclease Cas2 [Haloarcula argentinensis] |
| 491106993 | WP_004966759.1 | CRISPR-associated endonuclease Cas2 [Haloarcula sinaiiensis] |
| 543414356 | GAD53618.1 | CRISPR-associated RecB family exonuclease Cas4b [Halarchaeum acidiphilum MH1-52-1] |
| 76558142 | CAI49729.1 | CRISPR-associated endonuclease Cas2 [Natronomonas pharaonis DSM 2160] |
| 76558141 | CAI49728.1 | CRISPR-associated endonuclease Cas1 [Natronomonas pharaonis DSM 2160] |
| 76558140 | CAI49727.1 | CRISPR-associated exonuclease Cas4 [Natronomonas pharaonis DSM 2160] |
| 76558135 | CAI49722.1 | CRISPR-associated endoribonuclease Cas6 [Natronomonas pharaonis DSM 2160] |
| 528526427 | CCQ33716.1 | CRISPR-associated helicase, Cas3 [Halorhabdus tiamatea SARL4B] |
| 556695521 | ESP86778.1 | CRISPR-associated endoribonuclease Cas6 [Candidatus Halobonum tyrrellensis G22] |
| 495010977 | WP_007736989.1 | CRISPR-associated DNA-binding Csa3 [Halococcus thailandensis] |
| 491182628 | WP_005040990.1 | CRISPR-associated DNA-binding Csa3 [Halococcus salifodinae] |
| 445806447 | EMA56576.1 | CRISPR-associated DNA-binding Csa3 [Halococcus thailandensis JCM 13552] |
| 445804444 | EMA54700.1 | CRISPR-associated DNA-binding Csa3 [Halococcus salifodinae DSM 8989] |
| 543413948 | GAD53847.1 | CRISPR-associated helicase Cas3 [Halarchaeum acidiphilum MH1-52-1] |
| 543413947 | GAD53846.1 | CRISPR-associated RecB family exonuclease Cas4a [Halarchaeum acidiphilum MH1-52-1] |
| 495010971 | WP_007736983.1 | CRISPR-associated helicase [Halococcus thailandensis] |
| 445806442 | EMA56571.1 | CRISPR-associated helicase [Halococcus thailandensis JCM 13552] |
| 573487043 | AHG05448.1 | helicase (plasmid) [Halobacterium sp. DL1] |
| 746356223 | WP_039400889.1 | helicase [haloarchaeon 3A1_DGR] |
| 564124629 | AHB67949.1 | helicase (plasmid) [Haloarcula hispanica N601] |
| 55229001 | AAV44424.1 | putative helicase (plasmid) [Haloarcula marismortui ATCC 43049] |
| 257171182 | ACV48941.1 | putative helicase [Halomicrobium mukohataei DSM 12286] |
| 256691290 | ACV11627.1 | putative helicase [Halorhabdus utahensis DSM 12940] |
| 517069340 | WP_018258158.1 | helicase [Halomicrobium katesii] |
| 503796721 | WP_014030715.1 | helicase [Haloarcula hispanica] |
| 495257577 | WP_007982332.1 | helicase [Haladaptatus paucihalophilus] |
| 506269426 | WP_015789201.1 | helicase [Halorhabdus utahensis] |
| 506244008 | WP_015763783.1 | helicase [Halomicrobium mukohataei] |
| 445756794 | EMA08152.1 | putative helicase [Haloarcula sinaiiensis ATCC 33800] |
| 320548911 | EFW90578.1 | putative helicase [Haladaptatus paucihalophilus DX253] |
| 494345543 | WP_007188930.1 | helicase [Haloarcula californiae] |
| 445806735 | EMA56846.1 | helicase [Halococcus thailandensis JCM 13552] |
| 445767706 | EMA18800.1 | helicase [Haloarcula californiae ATCC 33799] |
