# Supplementary material for: Determining virus-host interactions and glycerol metabolism profiles in geographically diverse solar salterns with metagenomics
Source: PeerJ. 2017 Jan 10;5:e2844. doi: 10.7717/peerj.2844 (PMC5228507; doi:10.7717/peerj.2844)
Supplement: Table S12 — The library was aligned against Newbler contigs assembled from the Cahuil/C34, combined Chula Vista, and combined Santa Pola/Isla Cristina metagenomes. [file peerj-05-2844-s019.docx]

Table S12: The library of *cas* genes identified in Haloferacales genomes that were aligned against Newbler contigs assembled from the Cahuil/C34, combined Chula Vista, and combined Santa Pola/Isla Cristina metagenomes

| GI | GB | Description |
| --- | --- | --- |
| 544612413 | WP_021050764.1 | CRISPR-associated protein [Haloquadratum walsbyi] |
| 222454589 | ACM58852.1 | CRISPR-associated protein, Csh2 family (plasmid) [Halorubrum lacusprofundi ATCC 49239] |
| 222454588 | ACM58851.1 | CRISPR-associated protein Cas5, Hmari subtype (plasmid) [Halorubrum lacusprofundi ATCC 49239] |
| 222454585 | ACM58848.1 | CRISPR-associated protein Cas1 (plasmid) [Halorubrum lacusprofundi ATCC 49239] |
| 222454584 | ACM58847.1 | CRISPR-associated protein Cas2 (plasmid) [Halorubrum lacusprofundi ATCC 49239] |
| 222454586 | ACM58849.1 | CRISPR-associated protein Cas4 (plasmid) [Halorubrum lacusprofundi ATCC 49239] |
| 222454823 | ACM59086.1 | CRISPR-associated protein Cas2 (plasmid) [Halorubrum lacusprofundi ATCC 49239] |
| 222454822 | ACM59085.1 | CRISPR-associated protein Cas1 (plasmid) [Halorubrum lacusprofundi ATCC 49239] |
| 222454821 | ACM59084.1 | CRISPR-associated protein Cas4 (plasmid) [Halorubrum lacusprofundi ATCC 49239] |
| 222454819 | ACM59082.1 | CRISPR-associated protein Csc1 (plasmid) [Halorubrum lacusprofundi ATCC 49239] |
| 222454818 | ACM59081.1 | CRISPR-associated protein Csc2 (plasmid) [Halorubrum lacusprofundi ATCC 49239] |
| 222454817 | ACM59080.1 | CRISPR-associated protein Csc3 (plasmid) [Halorubrum lacusprofundi ATCC 49239] |
| 222454591 | ACM58854.1 | CRISPR-associated protein Cas6 (plasmid) [Halorubrum lacusprofundi ATCC 49239] |
| 222454590 | ACM58853.1 | CRISPR-associated protein, Csh1 family (plasmid) [Halorubrum lacusprofundi ATCC 49239] |
| 756974975 | WP_042663236.1 | CRISPR-associated protein Cas3 [Haloferax sp. ATB1] |
| 388246497 | AFK21440.1 | CRISPR-associated Cas3 family protein (plasmid) [Haloferax mediterranei ATCC 33500] |
| 495599996 | WP_008324575.1 | CRISPR-associated Cas3 family protein [Haloferax elongans] |
| 445745777 | ELZ97243.1 | CRISPR-associated Cas3 family protein [Haloferax mediterranei ATCC 33500] |
| 445742165 | ELZ93661.1 | CRISPR-associated Cas3 family protein [Haloferax mucosum ATCC BAA-1512] |
| 445733001 | ELZ84576.1 | CRISPR-associated Cas3 family protein [Haloferax elongans ATCC BAA-1513] |
| 631806802 | AHZ24495.1 | CRISPR-associated protein Cas6 (plasmid) [Haloferax mediterranei ATCC 33500] |
| 631806801 | AHZ24494.1 | CRISPR-associated protein Csh1 (plasmid) [Haloferax mediterranei ATCC 33500] |
| 631806800 | AHZ24493.1 | CRISPR-associated protein Csh2 (plasmid) [Haloferax mediterranei ATCC 33500] |
| 631806799 | AHZ24492.1 | CRISPR-associated protein Cas5 (plasmid) [Haloferax mediterranei ATCC 33500] |
| 631806796 | AHZ24489.1 | CRISPR-associated protein Cas1 (plasmid) [Haloferax mediterranei ATCC 33500] |
| 631806795 | AHZ24488.1 | CRISPR-associated protein Cas2 (plasmid) [Haloferax mediterranei ATCC 33500] |
| 291369447 | ADE01675.1 | CRISPR-associated protein Cas6 (plasmid) [Haloferax volcanii DS2] |
| 495369681 | WP_008094394.1 | CRISPR-associated protein Cas6 [Haloferax prahovense] |
| 504368112 | WP_014555214.1 | CRISPR-associated protein Cas3 [Haloquadratum walsbyi] |
| 502800038 | WP_013035014.1 | CRISPR-associated protein Cas6 [Haloferax volcanii] |
| 490145995 | WP_004046325.1 | CRISPR-associated Cas3 family protein [Halorubrum saccharovorum] |
| 445717150 | ELZ68871.1 | CRISPR-associated protein Cas6 [Haloferax prahovense DSM 18310] |
| 445690551 | ELZ42761.1 | CRISPR-associated Cas3 family protein [Halorubrum saccharovorum DSM 1137] |
| 445577659 | ELY32091.1 | CRISPR-associated protein Cas6 [Haloferax volcanii DS2] |
| 432197975 | ELK54311.1 | CRISPR-associated protein Cas5, Hmari subtype [Haloferax sp. BAB2207] |
| 432197974 | ELK54310.1 | Csh2 family CRISPR-associated protein [Haloferax sp. BAB2207] |
| 432197972 | ELK54308.1 | CRISPR-associated protein Cas6 [Haloferax sp. BAB2207] |
| 631806797 | AHZ24490.1 | CRISPR-associated protein Cas4 (plasmid) [Haloferax mediterranei ATCC 33500] |
| 504368108 | WP_014555210.1 | CRISPR-associated protein Cas6 [Haloquadratum walsbyi] |
| 445819508 | EMA69349.1 | CRISPR-associated protein Cas2 [Halorubrum kocurii JCM 14978] |
| 445819507 | EMA69348.1 | CRISPR-associated protein Cas1 [Halorubrum kocurii JCM 14978] |
| 445819504 | EMA69345.1 | CRISPR-associated protein Cas5, Hmari subtype [Halorubrum kocurii JCM 14978] |
| 445819503 | EMA69344.1 | CRISPR-associated protein, Csh2 family [Halorubrum kocurii JCM 14978] |
| 445819501 | EMA69342.1 | CRISPR-associated protein Cas6 [Halorubrum kocurii JCM 14978] |
| 445748297 | ELZ99745.1 | CRISPR-associated protein Cas2 [Haloferax denitrificans ATCC 35960] |
| 445748296 | ELZ99744.1 | CRISPR-associated protein Cas1 [Haloferax denitrificans ATCC 35960] |
| 445748293 | ELZ99741.1 | CRISPR-associated protein Cas5, Hmari subtype [Haloferax denitrificans ATCC 35960] |
| 445748292 | ELZ99740.1 | CRISPR-associated protein, Csh2 family [Haloferax denitrificans ATCC 35960] |
| 445748290 | ELZ99738.1 | CRISPR-associated protein Cas6 [Haloferax denitrificans ATCC 35960] |
| 445745781 | ELZ97247.1 | CRISPR-associated Cas6 family protein [Haloferax mediterranei ATCC 33500] |
| 445745779 | ELZ97245.1 | CRISPR-associated Csh2 family protein [Haloferax mediterranei ATCC 33500] |
| 445745778 | ELZ97244.1 | CRISPR-associated Cas5h family protein [Haloferax mediterranei ATCC 33500] |
| 445745775 | ELZ97241.1 | CRISPR-associated protein Cas1 [Haloferax mediterranei ATCC 33500] |
| 445745774 | ELZ97240.1 | CRISPR-associated Cas2 family protein [Haloferax mediterranei ATCC 33500] |
| 445744554 | ELZ96029.1 | CRISPR-associated protein Cas2 [Haloferax sulfurifontis ATCC BAA-897] |
| 445744553 | ELZ96028.1 | CRISPR-associated Cas1 family protein [Haloferax sulfurifontis ATCC BAA-897] |
| 445742970 | ELZ94458.1 | CRISPR-associated Cas1 family protein [Haloferax alexandrinus JCM 10717] |
| 445742969 | ELZ94457.1 | CRISPR-associated protein Cas2 [Haloferax alexandrinus JCM 10717] |
| 445742168 | ELZ93664.1 | CRISPR-associated Cas2 family protein [Haloferax mucosum ATCC BAA-1512] |
| 445742167 | ELZ93663.1 | CRISPR-associated protein Cas1 [Haloferax mucosum ATCC BAA-1512] |
| 445742163 | ELZ93659.1 | CRISPR-associated Csh2 family protein [Haloferax mucosum ATCC BAA-1512] |
| 445742161 | ELZ93657.1 | CRISPR-associated Cas6 family protein [Haloferax mucosum ATCC BAA-1512] |
| 445737820 | ELZ89351.1 | CRISPR-associated protein, Csh2 family [Haloferax alexandrinus JCM 10717] |
| 445737819 | ELZ89350.1 | CRISPR-associated protein Cas5, Hmari subtype [Haloferax alexandrinus JCM 10717] |
| 445737816 | ELZ89347.1 | CRISPR-associated protein Cas1 [Haloferax alexandrinus JCM 10717] |
| 445737815 | ELZ89346.1 | CRISPR-associated protein Cas2 [Haloferax alexandrinus JCM 10717] |
| 445737434 | ELZ88968.1 | CRISPR-associated protein Cas6 [Haloferax alexandrinus JCM 10717] |
| 445733005 | ELZ84580.1 | CRISPR-associated Cas6 family protein [Haloferax elongans ATCC BAA-1513] |
| 445733003 | ELZ84578.1 | CRISPR-associated Csh2 family protein [Haloferax elongans ATCC BAA-1513] |
| 445732999 | ELZ84574.1 | CRISPR-associated protein Cas1 [Haloferax elongans ATCC BAA-1513] |
| 445732998 | ELZ84573.1 | CRISPR-associated Cas2 family protein [Haloferax elongans ATCC BAA-1513] |
| 445732888 | ELZ84466.1 | CRISPR-associated Cas5h family protein [Haloferax gibbonsii ATCC 33959] |
| 445732886 | ELZ84464.1 | CRISPR-associated protein Cas1 [Haloferax gibbonsii ATCC 33959] |
| 445732885 | ELZ84463.1 | CRISPR-associated protein Cas2 [Haloferax gibbonsii ATCC 33959] |
| 445732882 | ELZ84461.1 | CRISPR-associated protein Cas6 [Haloferax gibbonsii ATCC 33959] |
| 445724435 | ELZ76067.1 | CRISPR-associated protein Cas5, Hmari subtype [Haloferax lucentense DSM 14919] |
| 445724434 | ELZ76066.1 | Csh2 family CRISPR-associated protein [Haloferax lucentense DSM 14919] |
| 445724432 | ELZ76064.1 | CRISPR-associated protein Cas6 [Haloferax lucentense DSM 14919] |
| 445720591 | ELZ72264.1 | CRISPR-associated protein Cas6 [Haloferax sp. ATCC BAA-644] |
| 445720589 | ELZ72262.1 | CRISPR-associated protein, Csh2 family [Haloferax sp. ATCC BAA-644] |
| 445720588 | ELZ72261.1 | CRISPR-associated protein Cas5, Hmari subtype [Haloferax sp. ATCC BAA-644] |
| 445720585 | ELZ72258.1 | CRISPR-associated protein Cas1 [Haloferax sp. ATCC BAA-644] |
| 445720584 | ELZ72257.1 | CRISPR-associated protein Cas2 [Haloferax sp. ATCC BAA-644] |
| 445720377 | ELZ72051.1 | CRISPR-associated protein Cas6 [Haloferax lucentense DSM 14919] |
| 445720188 | ELZ71864.1 | CRISPR-associated protein Cas2 [Haloferax lucentense DSM 14919] |
| 445720187 | ELZ71863.1 | CRISPR-associated protein Cas1 [Haloferax lucentense DSM 14919] |
| 445720184 | ELZ71860.1 | CRISPR-associated protein Cas5, Hmari subtype [Haloferax lucentense DSM 14919] |
| 445720183 | ELZ71859.1 | CRISPR-associated protein, Csh2 family [Haloferax lucentense DSM 14919] |
| 445717152 | ELZ68873.1 | CRISPR-associated Csh2 family protein [Haloferax prahovense DSM 18310] |
| 445717148 | ELZ68869.1 | CRISPR-associated protein Cas1 [Haloferax prahovense DSM 18310] |
| 445717147 | ELZ68868.1 | CRISPR-associated Cas2 family protein [Haloferax prahovense DSM 18310] |
| 445712009 | ELZ63794.1 | CRISPR-associated protein Cas6 [Haloferax sp. ATCC BAA-644] |
| 445712007 | ELZ63792.1 | Csh2 family CRISPR-associated protein [Haloferax sp. ATCC BAA-644] |
| 445712006 | ELZ63791.1 | CRISPR-associated protein Cas5 [Haloferax sp. ATCC BAA-644] |
| 445710854 | ELZ62650.1 | CRISPR-associated protein Cas5 [Haloferax sp. ATCC BAA-645] |
| 445710853 | ELZ62649.1 | Csh2 family CRISPR-associated protein [Haloferax sp. ATCC BAA-645] |
| 445710851 | ELZ62647.1 | CRISPR-associated protein Cas6 [Haloferax sp. ATCC BAA-645] |
| 445708593 | ELZ60432.1 | CRISPR-associated protein Cas2 [Haloferax sp. ATCC BAA-645] |
| 445708592 | ELZ60431.1 | CRISPR-associated protein Cas1 [Haloferax sp. ATCC BAA-645] |
| 445708589 | ELZ60428.1 | CRISPR-associated protein Cas5, Hmari subtype [Haloferax sp. ATCC BAA-645] |
| 445708588 | ELZ60427.1 | CRISPR-associated protein, Csh2 family [Haloferax sp. ATCC BAA-645] |
| 445708586 | ELZ60425.1 | CRISPR-associated protein Cas6 [Haloferax sp. ATCC BAA-645] |
| 445707690 | ELZ59543.1 | CRISPR-associated protein Cas2 [Haloferax sp. ATCC BAA-646] |
| 445707689 | ELZ59542.1 | CRISPR-associated protein Cas1 [Haloferax sp. ATCC BAA-646] |
| 445707686 | ELZ59539.1 | CRISPR-associated protein Cas5, Hmari subtype [Haloferax sp. ATCC BAA-646] |
| 445707685 | ELZ59538.1 | CRISPR-associated protein, Csh2 family [Haloferax sp. ATCC BAA-646] |
| 445707683 | ELZ59536.1 | CRISPR-associated protein Cas6 [Haloferax sp. ATCC BAA-646] |
| 445705979 | ELZ57866.1 | CRISPR-associated protein Cas5 [Haloferax sp. ATCC BAA-646] |
| 445705978 | ELZ57865.1 | Csh2 family CRISPR-associated protein [Haloferax sp. ATCC BAA-646] |
| 445705976 | ELZ57863.1 | CRISPR-associated protein Cas6 [Haloferax sp. ATCC BAA-646] |
| 445691207 | ELZ43399.1 | CRISPR-associated protein Cas5, Hmari subtype [Halorubrum coriense DSM 10284] |
| 445691206 | ELZ43398.1 | CRISPR-associated protein, Csh2 family [Halorubrum coriense DSM 10284] |
| 445691203 | ELZ43395.1 | CRISPR-associated protein Cas6 [Halorubrum coriense DSM 10284] |
| 445690555 | ELZ42765.1 | CRISPR-associated protein Cas6 [Halorubrum saccharovorum DSM 1137] |
| 445690553 | ELZ42763.1 | Csh2 family CRISPR-associated protein [Halorubrum saccharovorum DSM 1137] |
| 445690549 | ELZ42759.1 | CRISPR-associated protein Cas1 [Halorubrum saccharovorum DSM 1137] |
| 445690548 | ELZ42758.1 | CRISPR-associated protein Cas2 [Halorubrum saccharovorum DSM 1137] |
| 445688495 | ELZ40752.1 | CRISPR-associated protein Cas6 [Halorubrum californiensis DSM 19288] |
| 445688493 | ELZ40750.1 | CRISPR-associated protein, Csh2 family [Halorubrum californiensis DSM 19288] |
| 445688492 | ELZ40749.1 | CRISPR-associated protein Cas5, Hmari subtype [Halorubrum californiensis DSM 19288] |
| 445688489 | ELZ40746.1 | CRISPR-associated protein Cas1 [Halorubrum californiensis DSM 19288] |
| 445688488 | ELZ40745.1 | CRISPR-associated protein Cas2 [Halorubrum californiensis DSM 19288] |
| 445577667 | ELY32099.1 | CRISPR-associated protein Cas2 [Haloferax volcanii DS2] |
| 445577666 | ELY32098.1 | CRISPR-associated protein Cas1 [Haloferax volcanii DS2] |
| 445577662 | ELY32094.1 | CRISPR-associated protein Cas5, Hmari subtype [Haloferax volcanii DS2] |
| 445577661 | ELY32093.1 | CRISPR-associated protein, Csh2 family [Haloferax volcanii DS2] |
| 496122562 | WP_008847069.1 | CRISPR-associated protein Cas5 [Halorubrum kocurii] |
| 495847873 | WP_008572452.1 | MULTISPECIES: CRISPR-associated protein Cas5 [Haloferax] |
| 495720289 | WP_008444868.1 | CRISPR-associated protein Cas5 [Halorubrum californiense] |
| 495369687 | WP_008094400.1 | CRISPR-associated protein Cas5 [Haloferax prahovense] |
| 493057808 | WP_006114886.1 | CRISPR-associated protein Cas5 [Halorubrum coriense] |
| 491114036 | WP_004972493.1 | CRISPR-associated protein Cas5 [Haloferax gibbonsii] |
| 491112827 | WP_004971286.1 | CRISPR-associated protein Cas5 [Haloferax denitrificans] |
| 490166027 | WP_004064674.1 | MULTISPECIES: CRISPR-associated protein Cas5 [Haloferax] |
| 490163813 | WP_004062466.1 | MULTISPECIES: CRISPR-associated protein Cas5 [Haloferax] |
| 490145996 | WP_004046326.1 | CRISPR-associated protein Cas5 [Halorubrum saccharovorum] |
| 811258927 | CQR53165.1 | CRISPR-associated protein (Cas_Cas5) [Haloferax sp. Arc-Hr] |
| 811258925 | CQR53160.1 | CRISPR-associated protein (cas_TM1802) [Haloferax sp. Arc-Hr] |
| 339729023 | CCC40224.1 | CRISPR-associated protein Cas7 [Haloquadratum walsbyi C23] |
| 339729022 | CCC40223.1 | CRISPR-associated protein Cas8b [Haloquadratum walsbyi C23] |
| 339728196 | CCC39329.1 | CRISPR-associated protein Csc1 [Haloquadratum walsbyi C23] |
| 339728195 | CCC39328.1 | CRISPR-associated protein Csc2 [Haloquadratum walsbyi C23] |
| 339728194 | CCC39327.1 | CRISPR-associated protein Cas10d [Haloquadratum walsbyi C23] |
| 339728192 | CCC39325.1 | CRISPR-associated DNA-binding protein Csm6 [Haloquadratum walsbyi C23] |
| 756974974 | WP_042663235.1 | CRISPR-associated protein Cas5 [Haloferax sp. ATB1] |
| 756974962 | WP_042663224.1 | CRISPR-associated protein Csh2 [Haloferax sp. ATB1] |
| 756974958 | WP_042663221.1 | CRISPR-associated protein Cas6 [Haloferax sp. ATB1] |
| 756973645 | WP_042661972.1 | CRISPR-associated protein Csh2 [Haloferax sp. ATB1] |
| 756973643 | WP_042661970.1 | CRISPR-associated protein Cas6 [Haloferax sp. ATB1] |
| 756973641 | WP_042661968.1 | CRISPR-associated protein Cas1 [Haloferax sp. ATB1] |
| 388246500 | AFK21443.1 | CRISPR-associated Cas2 family protein (plasmid) [Haloferax mediterranei ATCC 33500] |
| 388246499 | AFK21442.1 | CRISPR-associated Cas1 family protein (plasmid) [Haloferax mediterranei ATCC 33500] |
| 388246496 | AFK21439.1 | CRISPR-associated Cas5h family protein (plasmid) [Haloferax mediterranei ATCC 33500] |
| 388246495 | AFK21438.1 | CRISPR-associated Csh2 family protein (plasmid) [Haloferax mediterranei ATCC 33500] |
| 388246494 | AFK21437.1 | CRISPR-associated Csh1 family protein (plasmid) [Haloferax mediterranei ATCC 33500] |
| 388246493 | AFK21436.1 | CRISPR-associated Cas6 family protein (plasmid) [Haloferax mediterranei ATCC 33500] |
| 291369979 | ADE02207.1 | CRISPR-associated protein Cas2 (plasmid) [Haloferax volcanii DS2] |
| 291369715 | ADE01943.1 | CRISPR-associated protein Cas5, Hmari subtype (plasmid) [Haloferax volcanii DS2] |
| 291369627 | ADE01855.1 | CRISPR-associated protein, Csh2 family (plasmid) [Haloferax volcanii DS2] |
| 291369516 | ADE01744.1 | CRISPR-associated protein Cas1 (plasmid) [Haloferax volcanii DS2] |
| 544612414 | WP_021050765.1 | CRISPR-associated protein Cas7/Csh2, subtype I-B/HMARI [Haloquadratum walsbyi] |
| 541182738 | ERG91248.1 | CRISPR-associated protein Cas5, subtype I-B/HMARI [Haloquadratum walsbyi J07HQW1] |
| 541182737 | ERG91247.1 | CRISPR-associated protein Cas7/Csh2, subtype I-B/HMARI [Haloquadratum walsbyi J07HQW1] |
| 541182736 | ERG91246.1 | CRISPR-associated protein TM1802 (cas_TM1802) [Haloquadratum walsbyi J07HQW1] |
| 491114040 | WP_004972497.1 | CRISPR-associated protein Cas6 [Haloferax gibbonsii] |
| 491112830 | WP_004971289.1 | CRISPR-associated protein Cas1 [Haloferax denitrificans] |
| 491112826 | WP_004971285.1 | CRISPR-associated protein, Csh2 family [Haloferax denitrificans] |
| 491112824 | WP_004971283.1 | CRISPR-associated protein, Csh1 family [Haloferax denitrificans] |
| 491112822 | WP_004971281.1 | CRISPR-associated protein Cas6 [Haloferax denitrificans] |
| 495852334 | WP_008576913.1 | MULTISPECIES: CRISPR-associated protein Cas5 [Haloferax] |
| 495847879 | WP_008572458.1 | MULTISPECIES: CRISPR-associated protein Cas6 [Haloferax] |
| 495847877 | WP_008572456.1 | MULTISPECIES: CRISPR-associated protein, Csh1 family [Haloferax] |
| 495847875 | WP_008572454.1 | MULTISPECIES: CRISPR-associated protein, Csh2 family [Haloferax] |
| 495847867 | WP_008572446.1 | MULTISPECIES: CRISPR-associated protein Cas1 [Haloferax] |
| 493648623 | WP_006600194.1 | MULTISPECIES: CRISPR-associated protein Cas4 [Haloferax] |
| 490166014 | WP_004064661.1 | MULTISPECIES: CRISPR-associated protein Cas6 [Haloferax] |
| 490163810 | WP_004062463.1 | MULTISPECIES: CRISPR-associated protein Cas6 [Haloferax] |
| 496122561 | WP_008847068.1 | CRISPR-associated protein, Csh2 family [Halorubrum kocurii] |
| 496122560 | WP_008847067.1 | CRISPR-associated protein, Csh1 family [Halorubrum kocurii] |
| 496122559 | WP_008847066.1 | CRISPR-associated protein Cas6 [Halorubrum kocurii] |
| 495720296 | WP_008444875.1 | CRISPR-associated protein Cas6 [Halorubrum californiense] |
| 495720294 | WP_008444873.1 | CRISPR-associated protein, Csh1 family [Halorubrum californiense] |
| 495720292 | WP_008444871.1 | CRISPR-associated protein, Csh2 family [Halorubrum californiense] |
| 495720281 | WP_008444860.1 | CRISPR-associated protein Cas1 [Halorubrum californiense] |
| 495599998 | WP_008324577.1 | CRISPR-associated Csh2 family protein [Haloferax elongans] |
| 495599994 | WP_008324573.1 | CRISPR-associated protein Cas1 [Haloferax elongans] |
| 495596107 | WP_008320686.1 | CRISPR-associated protein Cas1 [Haloferax mucosum] |
| 495596102 | WP_008320681.1 | CRISPR-associated Csh2 family protein [Haloferax mucosum] |
| 495596101 | WP_008320680.1 | CRISPR-associated Csh1 family protein [Haloferax mucosum] |
| 495369685 | WP_008094398.1 | CRISPR-associated Csh2 family protein [Haloferax prahovense] |
| 495369683 | WP_008094396.1 | CRISPR-associated protein Csh1 [Haloferax prahovense] |
| 494484860 | WP_007274333.1 | CRISPR-associated Cas1 family protein [Haloferax sulfurifontis] |
| 494484857 | WP_007274330.1 | CRISPR-associated protein Csc2 [Haloferax sulfurifontis] |
| 493649497 | WP_006601038.1 | CRISPR-associated protein, Csh1 family, partial [Haloferax alexandrinus] |
| 493649496 | WP_006601037.1 | CRISPR-associated protein, Csh2 family [Haloferax alexandrinus] |
| 493649495 | WP_006601036.1 | CRISPR-associated protein Cas1 [Haloferax alexandrinus] |
| 493648626 | WP_006600197.1 | CRISPR-associated protein Csc2 [Haloferax alexandrinus] |
| 493648622 | WP_006600193.1 | CRISPR-associated Cas1 family protein [Haloferax alexandrinus] |
| 493057806 | WP_006114885.1 | CRISPR-associated protein, Csh2 family [Halorubrum coriense] |
| 504545715 | WP_014732817.1 | CRISPR-associated protein Cas6 [Haloferax mediterranei] |
| 502800142 | WP_013035118.1 | CRISPR-associated protein Cas5 [Haloferax volcanii] |
| 490162157 | WP_004060812.1 | CRISPR-associated protein Csh1 [Haloferax mediterranei] |
| 490162156 | WP_004060811.1 | CRISPR-associated protein Csh2 [Haloferax mediterranei] |
| 490162155 | WP_004060810.1 | CRISPR-associated protein Cas5 [Haloferax mediterranei] |
| 490142785 | WP_004043127.1 | CRISPR-associated protein Csh2 [Haloferax volcanii] |
| 501864844 | WP_012660271.1 | CRISPR-associated protein Cas6 [Halorubrum lacusprofundi] |
| 501864461 | WP_012660066.1 | CRISPR-associated protein Csh2 [Halorubrum lacusprofundi] |
| 501864457 | WP_012660065.1 | CRISPR-associated protein Cas5 [Halorubrum lacusprofundi] |
| 504368806 | WP_014555908.1 | CRISPR-associated protein Cas1 [Haloquadratum walsbyi] |
| 504368802 | WP_014555904.1 | CRISPR-associated protein Csh2 [Haloquadratum walsbyi] |
| 504368801 | WP_014555903.1 | CRISPR-associated protein Csh1 [Haloquadratum walsbyi] |
| 504368114 | WP_014555216.1 | CRISPR-associated protein Cas1 [Haloquadratum walsbyi] |
| 504368113 | WP_014555215.1 | CRISPR-associated protein Cas4 [Haloquadratum walsbyi] |
| 504368111 | WP_014555213.1 | CRISPR-associated protein Csc1 [Haloquadratum walsbyi] |
| 504368110 | WP_014555212.1 | CRISPR-associated protein Csc2 [Haloquadratum walsbyi] |
| 504368109 | WP_014555211.1 | CRISPR-associated protein Csc3 [Haloquadratum walsbyi] |
| 502800067 | WP_013035043.1 | CRISPR-associated protein Cas1 [Haloferax volcanii] |
| 501864850 | WP_012660277.1 | CRISPR-associated protein Cas1 [Halorubrum lacusprofundi] |
| 501864846 | WP_012660273.1 | CRISPR-associated protein Csc2 [Halorubrum lacusprofundi] |
| 501864845 | WP_012660272.1 | CRISPR-associated protein Csc3 [Halorubrum lacusprofundi] |
| 501864444 | WP_012660062.1 | CRISPR-associated protein Cas1 [Halorubrum lacusprofundi] |
| 490166030 | WP_004064677.1 | CRISPR-associated protein Cas1 [Haloferax lucentense] |
| 490166026 | WP_004064673.1 | CRISPR-associated protein, Csh2 family [Haloferax lucentense] |
| 490166015 | WP_004064662.1 | CRISPR-associated protein, Csh1 family, partial [Haloferax lucentense] |
| 490162152 | WP_004060807.1 | CRISPR-associated protein Cas1 [Haloferax mediterranei] |
| 490145999 | WP_004046329.1 | CRISPR-associated protein Cas6 [Halorubrum saccharovorum] |
| 490145998 | WP_004046328.1 | CRISPR-associated protein, Csh1 family [Halorubrum saccharovorum] |
| 490145993 | WP_004046323.1 | CRISPR-associated protein Cas1 [Halorubrum saccharovorum] |
| 445819502 | EMA69343.1 | CRISPR-associated protein, Csh1 family [Halorubrum kocurii JCM 14978] |
| 445748291 | ELZ99739.1 | CRISPR-associated protein, Csh1 family [Haloferax denitrificans ATCC 35960] |
| 445745780 | ELZ97246.1 | CRISPR-associated Csh1 family protein [Haloferax mediterranei ATCC 33500] |
| 445744552 | ELZ96027.1 | CRISPR-associated protein Cas4 [Haloferax sulfurifontis ATCC BAA-897] |
| 445744549 | ELZ96024.1 | CRISPR-associated protein Csc2 [Haloferax sulfurifontis ATCC BAA-897] |
| 445742974 | ELZ94462.1 | CRISPR-associated protein Csc2 [Haloferax alexandrinus JCM 10717] |
| 445742971 | ELZ94459.1 | CRISPR-associated protein Cas4 [Haloferax alexandrinus JCM 10717] |
| 445742164 | ELZ93660.1 | CRISPR-associated Cas5h family protein [Haloferax mucosum ATCC BAA-1512] |
| 445742162 | ELZ93658.1 | CRISPR-associated Csh1 family protein [Haloferax mucosum ATCC BAA-1512] |
| 445737433 | ELZ88967.1 | CRISPR-associated protein, Csh1 family, partial [Haloferax alexandrinus JCM 10717] |
| 445733004 | ELZ84579.1 | CRISPR-associated Csh1 family protein [Haloferax elongans ATCC BAA-1513] |
| 445733002 | ELZ84577.1 | CRISPR-associated Cas5h family protein [Haloferax elongans ATCC BAA-1513] |
| 445732889 | ELZ84467.1 | CRISPR-associated protein, Csh2 family [Haloferax gibbonsii ATCC 33959] |
| 445720590 | ELZ72263.1 | CRISPR-associated protein, Csh1 family [Haloferax sp. ATCC BAA-644] |
| 445720378 | ELZ72052.1 | CRISPR-associated protein, Csh1 family, partial [Haloferax lucentense DSM 14919] |
| 445717153 | ELZ68874.1 | CRISPR-associated protein Cas5, Hmari subtype [Haloferax prahovense DSM 18310] |
| 445717151 | ELZ68872.1 | CRISPR-associated protein Csh1 [Haloferax prahovense DSM 18310] |
| 445708587 | ELZ60426.1 | CRISPR-associated protein, Csh1 family [Haloferax sp. ATCC BAA-645] |
| 445707684 | ELZ59537.1 | CRISPR-associated protein, Csh1 family [Haloferax sp. ATCC BAA-646] |
| 445690554 | ELZ42764.1 | CRISPR-associated protein, Csh1 family [Halorubrum saccharovorum DSM 1137] |
| 445690552 | ELZ42762.1 | CRISPR-associated protein Cas5, Hmari subtype [Halorubrum saccharovorum DSM 1137] |
| 445688494 | ELZ40751.1 | CRISPR-associated protein, Csh1 family [Halorubrum californiensis DSM 19288] |
| 491114037 | WP_004972494.1 | MULTISPECIES: CRISPR-associated protein, Csh2 family [Haloferax] |
| 910013508 | WP_049968221.1 | CRISPR-associated protein Cas4 [Haloferax alexandrinus] |
| 910013507 | WP_049968220.1 | CRISPR-associated protein Cas5 [Haloferax alexandrinus] |
| 910013503 | WP_049968216.1 | CRISPR-associated protein Cas6 [Haloferax alexandrinus] |
| 909691407 | WP_049934655.1 | CRISPR-associated protein Cas2 [Haloplanus natans] |
| 909691405 | WP_049934653.1 | CRISPR-associated protein Cas1 [Haloplanus natans] |
| 909691404 | WP_049934652.1 | CRISPR-associated protein Cas4 [Haloplanus natans] |
| 909691402 | WP_049934650.1 | CRISPR-associated protein Cas5 [Haloplanus natans] |
| 909691400 | WP_049934648.1 | CRISPR-associated protein Csh2 [Haloplanus natans] |
| 909691398 | WP_049934646.1 | CRISPR-associated protein Csh1 [Haloplanus natans] |
| 909691396 | WP_049934644.1 | CRISPR-associated protein Cas6 [Haloplanus natans] |
| 909690778 | WP_049934026.1 | CRISPR-associated protein Cas4 [Halorubrum lacusprofundi] |
| 909690768 | WP_049934016.1 | CRISPR-associated protein Csh1 [Halorubrum lacusprofundi] |
| 909690668 | WP_049933916.1 | CRISPR-associated protein Cas6 [Halorubrum lacusprofundi] |
| 909689303 | WP_049932648.1 | CRISPR-associated protein Cas3 [Halorubrum ezzemoulense] |
| 909689301 | WP_049932646.1 | CRISPR-associated protein Csh2 [Halorubrum ezzemoulense] |
| 909689161 | WP_049932506.1 | CRISPR-associated protein Cas5 [Halorubrum ezzemoulense] |
| 909689158 | WP_049932503.1 | CRISPR-associated protein Cas6 [Halorubrum ezzemoulense] |
| 909678745 | WP_049922099.1 | CRISPR-associated protein Csa3 [Haloferax sp. BAB2207] |
| 909674905 | WP_049918259.1 | CRISPR-associated protein Cas4 [Haloferax denitrificans] |
| 909674211 | WP_049917565.1 | CRISPR-associated protein Cas4 [Haloferax mediterranei] |
| 909674210 | WP_049917564.1 | CRISPR-associated protein Cas2 [Haloferax mediterranei] |
| 909672422 | WP_049915776.1 | CRISPR-associated protein Cas2 [Haloferax mucosum] |
| 909672417 | WP_049915771.1 | CRISPR-associated protein Cas5 [Haloferax mucosum] |
| 909672415 | WP_049915769.1 | CRISPR-associated protein Cas6 [Haloferax mucosum] |
| 909670166 | WP_049913520.1 | CRISPR-associated protein Cas4 [Haloferax prahovense] |
| 909670152 | WP_049913506.1 | CRISPR-associated protein Cas1 [Haloferax prahovense] |
| 909668482 | WP_049911836.1 | CRISPR-associated protein Cas1 [Halorubrum kocurii] |
| 909665569 | WP_049908923.1 | CRISPR-associated protein Cas2 [Halorubrum saccharovorum] |
| 909664318 | WP_049907672.1 | CRISPR-associated protein Csh1 [Haloferax elongans] |
| 909664317 | WP_049907671.1 | CRISPR-associated protein Cas2 [Haloferax elongans] |
| 909664289 | WP_049907643.1 | CRISPR-associated protein Cas6 [Haloferax elongans] |
| 909664288 | WP_049907642.1 | CRISPR-associated protein Cas5 [Haloferax elongans] |
| 909661582 | WP_049904936.1 | CRISPR-associated protein Cas1 [Haloferax gibbonsii] |
| 909661490 | WP_049904844.1 | CRISPR-associated protein Csa3 [Haloferax gibbonsii] |
| 909653668 | WP_049897081.1 | MULTISPECIES: CRISPR-associated protein Cas6 [Haloferax] |
| 909648857 | WP_049892270.1 | CRISPR-associated protein Cas5 [Haloquadratum walsbyi] |
| 909648358 | WP_049891771.1 | CRISPR-associated protein Cas6 [Haloquadratum walsbyi] |
| 756973642 | WP_042661969.1 | CRISPR-associated protein Cas4 [Haloferax sp. ATB1] |
| 388246498 | AFK21441.1 | CRISPR-associated Cas4 family protein (plasmid) [Haloferax mediterranei ATCC 33500] |
| 291369910 | ADE02138.1 | CRISPR-associated protein Cas4 (plasmid) [Haloferax volcanii DS2] |
| 495847869 | WP_008572448.1 | MULTISPECIES: CRISPR-associated protein Cas4 [Haloferax] |
| 490166029 | WP_004064676.1 | MULTISPECIES: CRISPR-associated protein Cas4 [Haloferax] |
| 496122564 | WP_008847071.1 | CRISPR-associated protein Cas4 [Halorubrum kocurii] |
| 495599995 | WP_008324574.1 | CRISPR-associated Cas4 family protein [Haloferax elongans] |
| 495596105 | WP_008320684.1 | CRISPR-associated Cas4 family protein [Haloferax mucosum] |
| 490162153 | WP_004060808.1 | CRISPR-associated protein Cas4 [Haloferax mediterranei] |
| 504368805 | WP_014555907.1 | CRISPR-associated protein Cas4 [Haloquadratum walsbyi] |
| 502800211 | WP_013035187.1 | CRISPR-associated protein Cas4 [Haloferax volcanii] |
| 501864445 | WP_012660063.1 | CRISPR-associated protein Cas4 [Halorubrum lacusprofundi] |
| 490145994 | WP_004046324.1 | CRISPR-associated protein Cas4 [Halorubrum saccharovorum] |
| 445819506 | EMA69347.1 | CRISPR-associated protein Cas4 [Halorubrum kocurii JCM 14978] |
| 445748295 | ELZ99743.1 | CRISPR-associated protein Cas4 [Haloferax denitrificans ATCC 35960] |
| 445745776 | ELZ97242.1 | CRISPR-associated Cas4 family protein [Haloferax mediterranei ATCC 33500] |
| 445742166 | ELZ93662.1 | CRISPR-associated Cas4 family protein [Haloferax mucosum ATCC BAA-1512] |
| 445737817 | ELZ89348.1 | CRISPR-associated protein Cas4 [Haloferax alexandrinus JCM 10717] |
| 445733000 | ELZ84575.1 | CRISPR-associated Cas4 family protein [Haloferax elongans ATCC BAA-1513] |
| 445720586 | ELZ72259.1 | CRISPR-associated protein Cas4 [Haloferax sp. ATCC BAA-644] |
| 445720186 | ELZ71862.1 | CRISPR-associated protein Cas4 [Haloferax lucentense DSM 14919] |
| 445720182 | ELZ71858.1 | CRISPR-associated protein, Csh1 family, partial [Haloferax lucentense DSM 14919] |
| 445717149 | ELZ68870.1 | CRISPR-associated protein Cas4 [Haloferax prahovense DSM 18310] |
| 445708591 | ELZ60430.1 | CRISPR-associated protein Cas4 [Haloferax sp. ATCC BAA-645] |
| 445707688 | ELZ59541.1 | CRISPR-associated protein Cas4 [Haloferax sp. ATCC BAA-646] |
| 445690550 | ELZ42760.1 | CRISPR-associated protein Cas4 [Halorubrum saccharovorum DSM 1137] |
| 445688490 | ELZ40747.1 | CRISPR-associated protein Cas4 [Halorubrum californiensis DSM 19288] |
| 445577664 | ELY32096.1 | CRISPR-associated protein Cas4 [Haloferax volcanii DS2] |
| 811258924 | CQR53158.1 | CRISPR associated protein Cas6 [Haloferax sp. Arc-Hr] |
| 765680567 | D4GQN5.1 | RecName: Full=CRISPR-associated protein Cas8b |
| 765680566 | D4GQN6.1 | RecName: Full=CRISPR-associated protein Cas7 |
| 765680563 | D4GQN7.1 | RecName: Full=CRISPR-associated protein Cas5 |
| 222454820 | ACM59083.1 | CRISPR-associated helicase, Cyano-type (plasmid) [Halorubrum lacusprofundi ATCC 49239] |
| 490736648 | WP_004598973.1 | hypothetical protein [Halorubrum distributum] |
| 339729025 | CCC40226.1 | CRISPR-associated helicase Cas3 [Haloquadratum walsbyi C23] |
| 291370021 | ADE02249.1 | CRISPR-associated helicase Cas3 (plasmid) [Haloferax volcanii DS2] |
| 291369495 | ADE01723.1 | conserved hypothetical protein (plasmid) [Haloferax volcanii DS2] |
| 491112828 | WP_004971287.1 | CRISPR-associated helicase Cas3 [Haloferax denitrificans] |
| 495852331 | WP_008576910.1 | MULTISPECIES: CRISPR-associated helicase Cas3 [Haloferax] |
| 495847871 | WP_008572450.1 | MULTISPECIES: CRISPR-associated helicase Cas3 [Haloferax] |
| 490166028 | WP_004064675.1 | MULTISPECIES: CRISPR-associated helicase Cas3 [Haloferax] |
| 504368804 | WP_014555906.1 | CRISPR-associated helicase Cas3 [Haloquadratum walsbyi] |
| 490142784 | WP_004043126.1 | hypothetical protein [Haloferax volcanii] |
| 445818153 | EMA68020.1 | hypothetical protein C462_15150 [Halorubrum arcis JCM 13916] |
| 445807291 | EMA57376.1 | hypothetical protein C470_13958 [Halorubrum litoreum JCM 13561] |
| 445748294 | ELZ99742.1 | CRISPR-associated helicase Cas3 [Haloferax denitrificans ATCC 35960] |
| 445737818 | ELZ89349.1 | CRISPR-associated helicase Cas3 [Haloferax alexandrinus JCM 10717] |
| 445720587 | ELZ72260.1 | CRISPR-associated helicase Cas3 [Haloferax sp. ATCC BAA-644] |
| 445720185 | ELZ71861.1 | CRISPR-associated helicase Cas3 [Haloferax lucentense DSM 14919] |
| 445712005 | ELZ63790.1 | CRISPR-associated helicase Cas3 [Haloferax sp. ATCC BAA-644] |
| 445710855 | ELZ62651.1 | CRISPR-associated helicase Cas3 [Haloferax sp. ATCC BAA-645] |
| 445708590 | ELZ60429.1 | CRISPR-associated helicase Cas3 [Haloferax sp. ATCC BAA-645] |
| 445707687 | ELZ59540.1 | CRISPR-associated helicase Cas3 [Haloferax sp. ATCC BAA-646] |
| 445705980 | ELZ57867.1 | CRISPR-associated helicase Cas3 [Haloferax sp. ATCC BAA-646] |
| 445704182 | ELZ56100.1 | hypothetical protein C466_04619 [Halorubrum distributum JCM 10118] |
| 445693246 | ELZ45405.1 | hypothetical protein C465_14140 [Halorubrum distributum JCM 9100] |
| 445577663 | ELY32095.1 | CRISPR-associated helicase Cas3 [Haloferax volcanii DS2] |
| 445577660 | ELY32092.1 | hypothetical protein C498_09706 [Haloferax volcanii DS2] |
| 494484859 | WP_007274332.1 | CRISPR-associated helicase Cyano-type [Haloferax sulfurifontis] |
| 493648624 | WP_006600195.1 | CRISPR-associated helicase Cyano-type [Haloferax alexandrinus] |
| 339728197 | CCC39330.1 | CRISPR-associated helicase Cas3 [Haloquadratum walsbyi C23] |
| 541184072 | ERG92582.1 | DEAD/DEAH box helicase domain protein, partial [Haloquadratum walsbyi J07HQW1] |
| 445744551 | ELZ96026.1 | CRISPR-associated helicase, Cyano-type [Haloferax sulfurifontis ATCC BAA-897] |
| 445742972 | ELZ94460.1 | CRISPR-associated helicase, Cyano-type [Haloferax alexandrinus JCM 10717] |
| 339728193 | CCC39326.1 | CRISPR-associated endoribonuclease Cas6 [Haloquadratum walsbyi C23] |
| 756974963 | WP_042663225.1 | CRISPR-associated endonuclease Cas2 [Haloferax sp. ATB1] |
| 756973640 | WP_042661967.1 | CRISPR-associated endonuclease Cas2 [Haloferax sp. ATB1] |
| 504368807 | WP_014555909.1 | CRISPR-associated endonuclease Cas2 [Haloquadratum walsbyi] |
| 504368115 | WP_014555217.1 | CRISPR-associated endonuclease Cas2 [Haloquadratum walsbyi] |
| 501864443 | WP_012660061.1 | CRISPR-associated endonuclease Cas2 [Halorubrum lacusprofundi] |
| 496122566 | WP_008847073.1 | CRISPR-associated endonuclease Cas2 [Halorubrum kocurii] |
| 495720279 | WP_008444858.1 | CRISPR-associated endonuclease Cas2 [Halorubrum californiense] |
| 495369676 | WP_008094389.1 | CRISPR-associated endonuclease Cas2 [Haloferax prahovense] |
| 491114032 | WP_004972490.1 | CRISPR-associated endonuclease Cas2 [Haloferax gibbonsii] |
| 491112831 | WP_004971290.1 | CRISPR-associated endonuclease Cas2 [Haloferax denitrificans] |
| 490166032 | WP_004064679.1 | MULTISPECIES: CRISPR-associated endonuclease Cas2 [Haloferax] |
| 490162151 | WP_004060806.1 | CRISPR-associated endonuclease Cas2 [Haloferax mediterranei] |
| 490142791 | WP_004043133.1 | CRISPR-associated endonuclease Cas2 [Haloferax volcanii] |
| 339729028 | CCC40229.1 | CRISPR-associated endonuclease Cas2 [Haloquadratum walsbyi C23] |
| 339729027 | CCC40228.1 | CRISPR-associated endonuclease Cas1 [Haloquadratum walsbyi C23] |
| 339729024 | CCC40225.1 | CRISPR-associated endoribonuclease Cas5, Hmari subtype [Haloquadratum walsbyi C23] |
| 339728200 | CCC39333.1 | CRISPR-associated endonuclease Cas2 [Haloquadratum walsbyi C23] |
| 339728199 | CCC39332.1 | CRISPR-associated endonuclease Cas1 [Haloquadratum walsbyi C23] |
| 339728198 | CCC39331.1 | CRISPR-associated exonuclease Cas4 [Haloquadratum walsbyi C23] |
| 544612416 | WP_021050767.1 | CRISPR-associated endonuclease Cas3-HD [Haloquadratum walsbyi] |
| 541182739 | ERG91249.1 | CRISPR-associated endonuclease Cas3-HD [Haloquadratum walsbyi J07HQW1] |
| 490733198 | WP_004595537.1 | CRISPR-associated DNA-binding Csa3 [Halorubrum distributum] |
| 504368107 | WP_014555209.1 | CRISPR-associated DNA-binding Csa3 [Haloquadratum walsbyi] |
| 445707019 | ELZ58886.1 | CRISPR-associated DNA-binding Csa3 [Halorubrum distributum JCM 10118] |
| 445700343 | ELZ52346.1 | CRISPR-associated DNA-binding Csa3 [Halorubrum distributum JCM 9100] |
| 432192782 | ELK49604.1 | CRISPR-associated DNA-binding Csa3 [Haloferax sp. BAB2207] |
| 811258931 | CQR53172.1 | CRISPR-associated endoribonuclease Cas2 [Haloferax sp. Arc-Hr] |
| 811258930 | CQR53171.1 | CRISPR-associated endonuclease Cas1 [Haloferax sp. Arc-Hr] |
| 339729026 | CCC40227.1 | CRISPR-associated exonuclease Cas4 [Haloquadratum walsbyi C23] |
| 445735348 | ELZ86898.1 | CRISPR-associated DNA-binding Csa3 [Haloferax gibbonsii ATCC 33959] |
| 765680561 | D4GQN8.1 | RecName: Full=CRISPR-associated nuclease/helicase Cas3 |
| 765680565 | D4GQN4.1 | RecName: Full=CRISPR-associated endoribonuclease Cas6 |
| 765680564 | I3RB26.1 | RecName: Full=CRISPR-associated endoribonuclease Cas6 |
| 765680560 | D4GQP1.1 | RecName: Full=CRISPR-associated endoribonuclease Cas2 |
| 765680562 | D4GQN9.1 | RecName: Full=CRISPR-associated exonuclease Cas4 |
| 765680559 | D4GQP0.1 | RecName: Full=CRISPR-associated endonuclease Cas1 |
| 631806798 | AHZ24491.1 | helicase (plasmid) [Haloferax mediterranei ATCC 33500] |
| 222454587 | ACM58850.1 | putative helicase (plasmid) [Halorubrum lacusprofundi ATCC 49239] |
| 493057810 | WP_006114887.1 | helicase [Halorubrum coriense] |
| 445691208 | ELZ43400.1 | helicase [Halorubrum coriense DSM 10284] |
| 496122563 | WP_008847070.1 | helicase [Halorubrum kocurii] |
| 495720287 | WP_008444866.1 | helicase [Halorubrum californiense] |
| 495369689 | WP_008094402.1 | helicase [Haloferax prahovense] |
| 490162154 | WP_004060809.1 | helicase [Haloferax mediterranei] |
| 490142787 | WP_004043129.1 | helicase [Haloferax volcanii] |
| 445819505 | EMA69346.1 | helicase [Halorubrum kocurii JCM 14978] |
| 445717154 | ELZ68875.1 | helicase [Haloferax prahovense DSM 18310] |
| 445688491 | ELZ40748.1 | helicase [Halorubrum californiensis DSM 19288] |
| 544601826 | WP_021040304.1 | superfamily II helicase archaea-specific [Halonotius sp. J07HN4] |
| 541198251 | ERH06414.1 | superfamily II helicase, archaea-specific [Halonotius sp. J07HN4] |
| 490163814 | WP_004062467.1 | helicase [Haloferax lucentense] |
| 445724436 | ELZ76068.1 | helicase [Haloferax lucentense DSM 14919] |
