# Supplementary material for: Determining virus-host interactions and glycerol metabolism profiles in geographically diverse solar salterns with metagenomics
Source: PeerJ. 2017 Jan 10;5:e2844. doi: 10.7717/peerj.2844 (PMC5228507; doi:10.7717/peerj.2844)
Supplement: Table S13 — The library was aligned against Newbler contigs assembled from the Cahuil/C34, combined Chula Vista, and combined Santa Pola/Isla Cristina metagenomes. [file peerj-05-2844-s020.docx]

Table S13: The library of *cas* genes identified in Natrialbales genomes that were aligned against Newbler contigs assembled from the Cahuil/C34, combined Chula Vista, and combined Santa Pola/Isla Cristina metagenomes

| GI | GB | Description |
| --- | --- | --- |
| 445783277 | EMA34110.1 | CRISPR-associated protein [Halobiforma nitratireducens JCM 10879] |
| 445652087 | ELZ04988.1 | CRISPR-associated protein [Natrialba chahannaoensis JCM 10990] |
| 445638626 | ELY91753.1 | CRISPR-associated protein [Natrialba hulunbeirensis JCM 10989] |
| 445619053 | ELY72600.1 | CRISPR-associated protein [Natronobacterium gregoryi SP2] |
| 633918735 | KDD77846.1 | CRISPR-associated protein, partial [Haloterrigena sp. H13] |
| 493724240 | WP_006673618.1 | CRISPR-associated protein [Halobiforma nitratireducens] |
| 493703361 | WP_006653095.1 | CRISPR-associated protein [Natrialba hulunbeirensis] |
| 493157409 | WP_006165795.1 | CRISPR-associated protein [Natrialba chahannaoensis] |
| 909685432 | WP_049928780.1 | CRISPR-associated protein [Halopiger sp. IIH3] |
| 429137566 | AFZ74577.1 | CRISPR-associated protein Cas5, subtype I-B/HMARI [Natronobacterium gregoryi SP2] |
| 429137565 | AFZ74576.1 | CRISPR-associated protein Cas7/Csh2, subtype I-B/HMARI [Natronobacterium gregoryi SP2] |
| 429137564 | AFZ74575.1 | CRISPR-associated protein Cas8b/Csh1, subtype I-B/HMARI [Natronobacterium gregoryi SP2] |
| 429137568 | AFZ74579.1 | CRISPR-associated protein Cas4 [Natronobacterium gregoryi SP2] |
| 445643588 | ELY96633.1 | CRISPR-associated protein Cas6 [Natrialba chahannaoensis JCM 10990] |
| 493161517 | WP_006168575.1 | CRISPR-associated protein Cas6 [Natrialba chahannaoensis] |
| 445783278 | EMA34111.1 | CRISPR-associated protein Cas5, Hmari subtype [Halobiforma nitratireducens JCM 10879] |
| 445783275 | EMA34108.1 | CRISPR-associated protein Cas6 [Halobiforma nitratireducens JCM 10879] |
| 445779236 | EMA30171.1 | CRISPR-associated protein Cas6 [Halobiforma lacisalsi AJ5] |
| 445779234 | EMA30169.1 | CRISPR-associated protein, Csh2 family [Halobiforma lacisalsi AJ5] |
| 445779233 | EMA30168.1 | CRISPR-associated protein Cas5, Hmari subtype [Halobiforma lacisalsi AJ5] |
| 445779230 | EMA30165.1 | CRISPR-associated protein Cas1 [Halobiforma lacisalsi AJ5] |
| 445779229 | EMA30164.1 | CRISPR-associated protein Cas2 [Halobiforma lacisalsi AJ5] |
| 445664215 | ELZ16934.1 | CRISPR-associated protein Cas6 [Haloterrigena thermotolerans DSM 11522] |
| 445664213 | ELZ16932.1 | Csh2 family CRISPR-associated protein [Haloterrigena thermotolerans DSM 11522] |
| 445664212 | ELZ16931.1 | CRISPR-associated protein Cas5 [Haloterrigena thermotolerans DSM 11522] |
| 445664209 | ELZ16928.1 | CRISPR-associated protein Cas1 [Haloterrigena thermotolerans DSM 11522] |
| 445664208 | ELZ16927.1 | CRISPR-associated protein Cas2 [Haloterrigena thermotolerans DSM 11522] |
| 445652092 | ELZ04993.1 | CRISPR-associated protein Cas2 [Natrialba chahannaoensis JCM 10990] |
| 445652091 | ELZ04992.1 | CRISPR-associated protein Cas1 [Natrialba chahannaoensis JCM 10990] |
| 445652088 | ELZ04989.1 | CRISPR-associated protein Cas5, Hmari subtype [Natrialba chahannaoensis JCM 10990] |
| 445652085 | ELZ04986.1 | CRISPR-associated protein Cas6 [Natrialba chahannaoensis JCM 10990] |
| 445644086 | ELY97118.1 | CRISPR-associated protein Csc2 [Natrialba asiatica DSM 12278] |
| 445644082 | ELY97114.1 | CRISPR-associated Cas1 family protein [Natrialba asiatica DSM 12278] |
| 445644081 | ELY97113.1 | CRISPR-associated protein Cas2 [Natrialba asiatica DSM 12278] |
| 445643586 | ELY96631.1 | CRISPR-associated protein, Csh2 family [Natrialba chahannaoensis JCM 10990] |
| 445643585 | ELY96630.1 | CRISPR-associated protein Cas5, Hmari subtype [Natrialba chahannaoensis JCM 10990] |
| 445643029 | ELY96085.1 | CRISPR-associated protein Cas2 [Natrialba hulunbeirensis JCM 10989] |
| 445643028 | ELY96084.1 | CRISPR-associated Cas1 family protein [Natrialba hulunbeirensis JCM 10989] |
| 445638628 | ELY91755.1 | CRISPR-associated protein Cas6 [Natrialba hulunbeirensis JCM 10989] |
| 445638625 | ELY91752.1 | CRISPR-associated protein Cas5, Hmari subtype [Natrialba hulunbeirensis JCM 10989] |
| 445638622 | ELY91749.1 | CRISPR-associated protein Cas1 [Natrialba hulunbeirensis JCM 10989] |
| 445638621 | ELY91748.1 | CRISPR-associated protein Cas2 [Natrialba hulunbeirensis JCM 10989] |
| 445630635 | ELY83896.1 | CRISPR-associated protein Cas2 [Natrinema gari JCM 14663] |
| 445630634 | ELY83895.1 | CRISPR-associated protein Cas1 [Natrinema gari JCM 14663] |
| 445630631 | ELY83892.1 | CRISPR-associated protein Cas5 [Natrinema gari JCM 14663] |
| 445630630 | ELY83891.1 | CRISPR-associated protein, Csh2 family [Natrinema gari JCM 14663] |
| 445630628 | ELY83889.1 | CRISPR-associated protein Cas6 [Natrinema gari JCM 14663] |
| 445628549 | ELY81855.1 | CRISPR-associated protein Cas2 [Natrinema pallidum DSM 3751] |
| 445628548 | ELY81854.1 | CRISPR-associated protein Cas1 [Natrinema pallidum DSM 3751] |
| 445620369 | ELY73870.1 | CRISPR-associated protein Cas6, partial [Natrinema pallidum DSM 3751] |
| 445620327 | ELY73832.1 | CRISPR-associated protein Cas5 [Natrinema pallidum DSM 3751] |
| 445619055 | ELY72602.1 | CRISPR-associated protein Cas6 [Natronobacterium gregoryi SP2] |
| 445619052 | ELY72599.1 | CRISPR-associated protein Cas5, Hmari subtype [Natronobacterium gregoryi SP2] |
| 445619049 | ELY72596.1 | CRISPR-associated protein Cas1 [Natronobacterium gregoryi SP2] |
| 445619048 | ELY72595.1 | CRISPR-associated protein Cas2 [Natronobacterium gregoryi SP2] |
| 445602000 | ELY55980.1 | CRISPR-associated regulatory protein, DevR family [Natronococcus amylolyticus DSM 10524] |
| 445601999 | ELY55979.1 | CRISPR-associated protein Cas5 [Natronococcus amylolyticus DSM 10524] |
| 445587219 | ELY41483.1 | CRISPR-associated protein Cas6 [Natronorubrum sulfidifaciens JCM 14089] |
| 445587217 | ELY41481.1 | CRISPR-associated protein, Csh2 family [Natronorubrum sulfidifaciens JCM 14089] |
| 445587216 | ELY41480.1 | CRISPR-associated protein Cas5, Hmari subtype [Natronorubrum sulfidifaciens JCM 14089] |
| 445587213 | ELY41477.1 | CRISPR-associated protein Cas1 [Natronorubrum sulfidifaciens JCM 14089] |
| 445587212 | ELY41476.1 | CRISPR-associated protein Cas2 [Natronorubrum sulfidifaciens JCM 14089] |
| 504676159 | WP_014863261.1 | MULTISPECIES: CRISPR-associated protein Cas1 [Natrinema] |
| 495727693 | WP_008452272.1 | MULTISPECIES: CRISPR-associated protein, Csh2 family [Natrinema] |
| 493698384 | WP_006648202.1 | MULTISPECIES: CRISPR-associated protein Cas1 [Haloterrigena] |
| 635282939 | KDE59361.1 | CRISPR-associated protein Cas5 [Halostagnicola sp. A56] |
| 635282938 | KDE59360.1 | CRISPR-associated protein Csh2 [Halostagnicola sp. A56] |
| 635282937 | KDE59359.1 | CRISPR-associated protein Csh1 [Halostagnicola sp. A56] |
| 635279523 | KDE56713.1 | CRISPR-associated protein Cas2 [Halostagnicola sp. A56] |
| 495440267 | WP_008164961.1 | CRISPR-associated protein Cas5 [Natronorubrum sulfidifaciens] |
| 493724241 | WP_006673619.1 | CRISPR-associated protein Cas5 [Halobiforma nitratireducens] |
| 493703360 | WP_006653094.1 | CRISPR-associated protein Cas5 [Natrialba hulunbeirensis] |
| 493161511 | WP_006168572.1 | CRISPR-associated protein Cas5 [Natrialba chahannaoensis] |
| 493157411 | WP_006165797.1 | CRISPR-associated protein Cas5 [Natrialba chahannaoensis] |
| 397681801 | AFO56178.1 | CRISPR-associated protein Cas2 [Natrinema sp. J7-2] |
| 397681800 | AFO56177.1 | CRISPR-associated protein Cas1 [Natrinema sp. J7-2] |
| 397681797 | AFO56174.1 | CRISPR-associated protein Cas5, Hmari subtype [Natrinema sp. J7-2] |
| 397681796 | AFO56173.1 | CRISPR-associated protein, Csh2 family [Natrinema sp. J7-2] |
| 397681795 | AFO56172.1 | CRISPR-associated protein, Csh1 family [Natrinema sp. J7-2] |
| 397681794 | AFO56171.1 | CRISPR-associated protein Cas6 [Natrinema sp. J7-2] |
| 495727701 | WP_008452280.1 | CRISPR-associated protein Cas1 [Natrinema gari] |
| 495727694 | WP_008452273.1 | CRISPR-associated protein Cas5 [Natrinema gari] |
| 495727689 | WP_008452268.1 | CRISPR-associated protein, Csh1 family [Natrinema gari] |
| 495727687 | WP_008452266.1 | CRISPR-associated protein Cas6 [Natrinema gari] |
| 495701826 | WP_008426405.1 | CRISPR-associated regulatory protein, DevR family [Natronococcus jeotgali] |
| 495697739 | WP_008422318.1 | CRISPR-associated regulatory protein, DevR family [Natronococcus jeotgali] |
| 495440272 | WP_008164966.1 | CRISPR-associated protein Cas6 [Natronorubrum sulfidifaciens] |
| 495440270 | WP_008164964.1 | CRISPR-associated protein, Csh1 family [Natronorubrum sulfidifaciens] |
| 495440269 | WP_008164963.1 | CRISPR-associated protein, Csh2 family [Natronorubrum sulfidifaciens] |
| 495440262 | WP_008164956.1 | CRISPR-associated protein Cas1 [Natronorubrum sulfidifaciens] |
| 493724239 | WP_006673617.1 | CRISPR-associated protein, Csh1 family [Halobiforma nitratireducens] |
| 493703363 | WP_006653097.1 | CRISPR-associated protein Cas6 [Natrialba hulunbeirensis] |
| 493703362 | WP_006653096.1 | CRISPR-associated protein, Csh1 family [Natrialba hulunbeirensis] |
| 493703358 | WP_006653092.1 | CRISPR-associated protein Cas1 [Natrialba hulunbeirensis] |
| 493701604 | WP_006651368.1 | CRISPR-associated protein Cas4 [Natrialba hulunbeirensis] |
| 493701601 | WP_006651365.1 | CRISPR-associated protein Csc2 [Natrialba hulunbeirensis] |
| 493698389 | WP_006648207.1 | CRISPR-associated protein, Csh1 family [Haloterrigena thermotolerans] |
| 493698387 | WP_006648205.1 | CRISPR-associated protein Cas5 [Haloterrigena thermotolerans] |
| 493196772 | WP_006186929.1 | CRISPR-associated protein Cas5 [Natrinema pallidum] |
| 493196770 | WP_006186927.1 | CRISPR-associated protein, Csh2 family, partial [Natrinema pallidum] |
| 493196767 | WP_006186924.1 | CRISPR-associated protein Cas6, partial [Natrinema pallidum] |
| 493161515 | WP_006168574.1 | CRISPR-associated protein, Csh1 family [Natrialba chahannaoensis] |
| 493161513 | WP_006168573.1 | CRISPR-associated protein, Csh2 family [Natrialba chahannaoensis] |
| 493157416 | WP_006165802.1 | CRISPR-associated protein Cas1 [Natrialba chahannaoensis] |
| 493157408 | WP_006165794.1 | CRISPR-associated protein, Csh1 family [Natrialba chahannaoensis] |
| 493157406 | WP_006165792.1 | CRISPR-associated protein Cas6 [Natrialba chahannaoensis] |
| 493051977 | WP_006111345.1 | CRISPR-associated protein Csc2 [Natrialba asiatica] |
| 493051973 | WP_006111341.1 | CRISPR-associated protein Cas4 [Natrialba asiatica] |
| 493051972 | WP_006111340.1 | CRISPR-associated Cas1 family protein [Natrialba asiatica] |
| 491743546 | WP_005576679.1 | CRISPR-associated protein Cas6 [Natronobacterium gregoryi] |
| 491711242 | WP_005557132.1 | CRISPR-associated regulatory protein, DevR family [Natronococcus amylolyticus] |
| 491711241 | WP_005557131.1 | CRISPR-associated protein Cas5 [Natronococcus amylolyticus] |
| 504676154 | WP_014863256.1 | CRISPR-associated protein Csh1 [Natrinema sp. J7-2] |
| 494240648 | WP_007143042.1 | CRISPR-associated protein Csh1 [Halobiforma lacisalsi] |
| 494240646 | WP_007143041.1 | CRISPR-associated protein Csh2 [Halobiforma lacisalsi] |
| 491743544 | WP_005576677.1 | CRISPR-associated protein Csh1 [Natronobacterium gregoryi] |
| 491743542 | WP_005576675.1 | CRISPR-associated protein Csh2 [Natronobacterium gregoryi] |
| 491743541 | WP_005576674.1 | CRISPR-associated protein Cas5 [Natronobacterium gregoryi] |
| 491743533 | WP_005576666.1 | CRISPR-associated protein Cas1 [Natronobacterium gregoryi] |
| 445783276 | EMA34109.1 | CRISPR-associated protein, Csh1 family [Halobiforma nitratireducens JCM 10879] |
| 445664214 | ELZ16933.1 | CRISPR-associated protein, Csh1 family [Haloterrigena thermotolerans DSM 11522] |
| 445652086 | ELZ04987.1 | CRISPR-associated protein, Csh1 family [Natrialba chahannaoensis JCM 10990] |
| 445644083 | ELY97115.1 | CRISPR-associated protein Cas4 [Natrialba asiatica DSM 12278] |
| 445643587 | ELY96632.1 | CRISPR-associated protein, Csh1 family [Natrialba chahannaoensis JCM 10990] |
| 445643027 | ELY96083.1 | CRISPR-associated protein Cas4 [Natrialba hulunbeirensis JCM 10989] |
| 445643024 | ELY96080.1 | CRISPR-associated protein Csc2 [Natrialba hulunbeirensis JCM 10989] |
| 445638627 | ELY91754.1 | CRISPR-associated protein, Csh1 family [Natrialba hulunbeirensis JCM 10989] |
| 445630629 | ELY83890.1 | CRISPR-associated protein, Csh1 family [Natrinema gari JCM 14663] |
| 445620326 | ELY73831.1 | CRISPR-associated protein, Csh2 family, partial [Natrinema pallidum DSM 3751] |
| 445619054 | ELY72601.1 | CRISPR-associated protein, Csh1 family [Natronobacterium gregoryi SP2] |
| 445608223 | ELY62080.1 | CRISPR-associated regulatory protein, DevR family [Natronococcus jeotgali DSM 18795] |
| 445601996 | ELY55976.1 | CRISPR-associated protein Cas4 [Natronococcus amylolyticus DSM 10524] |
| 445598724 | ELY52778.1 | CRISPR-associated regulatory protein, DevR family [Natronococcus jeotgali DSM 18795] |
| 445587218 | ELY41482.1 | CRISPR-associated protein, Csh1 family [Natronorubrum sulfidifaciens JCM 14089] |
| 910247548 | WP_050052211.1 | CRISPR-associated protein Cas2 [Halostagnicola sp. A56] |
| 910246009 | WP_050050672.1 | CRISPR-associated protein Cas4 [Halostagnicola sp. A56] |
| 910246007 | WP_050050670.1 | CRISPR-associated protein Cas5 [Halostagnicola sp. A56] |
| 910246006 | WP_050050669.1 | CRISPR-associated protein Csh2 [Halostagnicola sp. A56] |
| 910246005 | WP_050050668.1 | CRISPR-associated protein Csh1 [Halostagnicola sp. A56] |
| 504676158 | WP_014863260.1 | MULTISPECIES: CRISPR-associated protein Cas4 [Natrinema] |
| 910075582 | WP_049992249.1 | CRISPR-associated protein Cas6 [Halopiger salifodinae] |
| 910075581 | WP_049992248.1 | CRISPR-associated protein Csh1 [Halopiger salifodinae] |
| 910075580 | WP_049992247.1 | CRISPR-associated protein Csh2 [Halopiger salifodinae] |
| 910012337 | WP_049967071.1 | CRISPR-associated protein Csh1 [Haloterrigena jeotgali] |
| 910012336 | WP_049967070.1 | CRISPR-associated protein Csh2 [Haloterrigena jeotgali] |
| 910012335 | WP_049967069.1 | CRISPR-associated protein Cas5 [Haloterrigena jeotgali] |
| 910012333 | WP_049967067.1 | CRISPR-associated protein Cas4 [Haloterrigena jeotgali] |
| 909685435 | WP_049928783.1 | CRISPR-associated protein Cas2 [Halopiger sp. IIH3] |
| 909685434 | WP_049928782.1 | CRISPR-associated protein Cas1 [Halopiger sp. IIH3] |
| 909685433 | WP_049928781.1 | CRISPR-associated protein Cas4 [Halopiger sp. IIH3] |
| 909685430 | WP_049928778.1 | CRISPR-associated protein Csc2 [Halopiger sp. IIH3] |
| 909685429 | WP_049928777.1 | CRISPR-associated protein Cas6 [Halopiger sp. IIH3] |
| 909676875 | WP_049920229.1 | CRISPR-associated protein Cas6 [Halobiforma nitratireducens] |
| 909668993 | WP_049912347.1 | CRISPR-associated protein Cas1 [Natrialba hulunbeirensis] |
| 909668974 | WP_049912328.1 | CRISPR-associated protein Cas6 [Natrialba hulunbeirensis] |
| 909664492 | WP_049907846.1 | CRISPR-associated protein Cas1 [Natrinema pallidum] |
| 909662084 | WP_049905438.1 | MULTISPECIES: CRISPR-associated protein Cas6 [Haloterrigena] |
| 909656866 | WP_049900279.1 | CRISPR-associated protein Csh2 [Natrinema sp. J7-1] |
| 909656863 | WP_049900276.1 | CRISPR-associated protein Csh1 [Natrinema sp. J7-1] |
| 909644697 | WP_049888110.1 | MULTISPECIES: CRISPR-associated protein Cas5 [Natrinema] |
| 909644696 | WP_049888109.1 | MULTISPECIES: CRISPR-associated protein Cas6 [Natrinema] |
| 635282941 | KDE59363.1 | CRISPR-associated protein Cas4 [Halostagnicola sp. A56] |
| 397681799 | AFO56176.1 | CRISPR-associated protein Cas4 [Natrinema sp. J7-2] |
| 493157414 | WP_006165800.1 | MULTISPECIES: CRISPR-associated protein Cas4 [Natrialba] |
| 495727698 | WP_008452277.1 | CRISPR-associated protein Cas4 [Natrinema gari] |
| 495440264 | WP_008164958.1 | CRISPR-associated protein Cas4 [Natronorubrum sulfidifaciens] |
| 493698385 | WP_006648203.1 | CRISPR-associated protein Cas4 [Haloterrigena thermotolerans] |
| 493196997 | WP_006187151.1 | CRISPR-associated protein Cas4 [Natrinema pallidum] |
| 491743536 | WP_005576669.1 | CRISPR-associated protein Cas4 [Natronobacterium gregoryi] |
| 445779231 | EMA30166.1 | CRISPR-associated protein Cas4 [Halobiforma lacisalsi AJ5] |
| 445664210 | ELZ16929.1 | CRISPR-associated protein Cas4 [Haloterrigena thermotolerans DSM 11522] |
| 445652090 | ELZ04991.1 | CRISPR-associated protein Cas4 [Natrialba chahannaoensis JCM 10990] |
| 445638623 | ELY91750.1 | CRISPR-associated protein Cas4 [Natrialba hulunbeirensis JCM 10989] |
| 445630633 | ELY83894.1 | CRISPR-associated protein Cas4 [Natrinema gari JCM 14663] |
| 445619409 | ELY72948.1 | CRISPR-associated protein Cas4 [Natrinema pallidum DSM 3751] |
| 445619050 | ELY72597.1 | CRISPR-associated protein Cas4 [Natronobacterium gregoryi SP2] |
| 445587214 | ELY41478.1 | CRISPR-associated protein Cas4 [Natronorubrum sulfidifaciens JCM 14089] |
| 429137569 | AFZ74580.1 | CRISPR-associated endonuclease Cas1 [Natronobacterium gregoryi SP2] |
| 429137567 | AFZ74578.1 | CRISPR-associated endonuclease Cas3-HD [Natronobacterium gregoryi SP2] |
| 429137570 | AFZ74581.1 | CRISPR-associated endoribonuclease Cas2 [Natronobacterium gregoryi SP2] |
| 429137563 | AFZ74574.1 | CRISPR-associated endoribonuclease Cas6 [Natronobacterium gregoryi SP2] |
| 493182304 | WP_006180277.1 | hypothetical protein [Natrinema pellirubrum] |
| 433304520 | AGB30332.1 | hypothetical protein Natpe_0401 [Natrinema pellirubrum DSM 15624] |
| 495440266 | WP_008164960.1 | CRISPR-associated helicase Cas3 [Natronorubrum sulfidifaciens] |
| 445783279 | EMA34112.1 | CRISPR-associated helicase Cas3, partial [Halobiforma nitratireducens JCM 10879] |
| 445665560 | ELZ18236.1 | hypothetical protein C478_01220 [Haloterrigena thermotolerans DSM 11522] |
| 445652089 | ELZ04990.1 | CRISPR-associated helicase Cas3 [Natrialba chahannaoensis JCM 10990] |
| 445648836 | ELZ01784.1 | hypothetical protein C481_09697 [Natrialba asiatica DSM 12278] |
| 445625936 | ELY79288.1 | hypothetical protein C488_04817 [Natrinema pellirubrum DSM 15624] |
| 445619051 | ELY72598.1 | CRISPR-associated helicase Cas3 [Natronobacterium gregoryi SP2] |
| 445587215 | ELY41479.1 | CRISPR-associated helicase Cas3 [Natronorubrum sulfidifaciens JCM 14089] |
| 493051974 | WP_006111342.1 | CRISPR-associated helicase Cyano-type [Natrialba asiatica] |
| 493701603 | WP_006651367.1 | CRISPR-associated helicase [Natrialba hulunbeirensis] |
| 445644084 | ELY97116.1 | CRISPR-associated helicase, Cyano-type [Natrialba asiatica DSM 12278] |
| 445643026 | ELY96082.1 | CRISPR-associated helicase [Natrialba hulunbeirensis JCM 10989] |
| 445615998 | ELY69634.1 | DEAD/DEAH box helicase domain-containing protein [Natronobacterium gregoryi SP2] |
| 445611132 | ELY64892.1 | DEAD/DEAH box helicase domain protein [Natrinema versiforme JCM 10478] |
| 445601994 | ELY55974.1 | CRISPR-associated endoribonuclease Cas2 [Natronococcus amylolyticus DSM 10524] |
| 495727703 | WP_008452282.1 | MULTISPECIES: CRISPR-associated endonuclease Cas2 [Natrinema] |
| 493698383 | WP_006648201.1 | MULTISPECIES: CRISPR-associated endonuclease Cas2 [Haloterrigena] |
| 495440261 | WP_008164955.1 | CRISPR-associated endonuclease Cas2 [Natronorubrum sulfidifaciens] |
| 493701606 | WP_006651370.1 | CRISPR-associated endonuclease Cas2 [Natrialba hulunbeirensis] |
| 493191532 | WP_006184181.1 | CRISPR-associated endonuclease Cas2 [Natrinema pallidum] |
| 493157417 | WP_006165803.1 | MULTISPECIES: CRISPR-associated endonuclease Cas2 [Natrialba] |
| 493051970 | WP_006111338.1 | CRISPR-associated endonuclease Cas2 [Natrialba asiatica] |
| 491743530 | WP_005576663.1 | CRISPR-associated endonuclease Cas2 [Natronobacterium gregoryi] |
| 445642052 | ELY95123.1 | CRISPR-associated DNA-binding Csa3 [Natrialba hulunbeirensis JCM 10989] |
| 445638624 | ELY91751.1 | CRISPR-associated helicase Cas3 [Natrialba hulunbeirensis JCM 10989] |
| 505046736 | WP_015233838.1 | helicase [Natronobacterium gregoryi] |
| 493477301 | WP_006432253.1 | DEAD/DEAH box helicase [Natrinema versiforme] |
| 429135865 | AFZ72876.1 | DNA/RNA helicase, superfamily II [Natronobacterium gregoryi SP2] |
| 491745336 | WP_005578469.1 | DEAD/DEAH box helicase [Natronobacterium gregoryi] |
| 445779232 | EMA30167.1 | putative helicase [Halobiforma lacisalsi AJ5] |
